# Identification of the mitophagy-related diagnostic biomarkers in hepatocellular carcinoma based on machine learning algorithm and construction of prognostic model

**DOI:** 10.3389/fonc.2023.1132559

**Published:** 2023-03-01

**Authors:** Dao-yuan Tu, Jun Cao, Jie Zhou, Bing-bing Su, Shun-yi Wang, Guo-qing Jiang, Sheng-jie Jin, Chi Zhang, Rui Peng, Dou-sheng Bai

**Affiliations:** Department of Hepatobiliary Surgery, Clinical Medical College, Yangzhou University, Yangzhou, Jiangsu, China

**Keywords:** mitophagy, machine learning, time, prognostic model, bioinformatics, hepatocelluar carcinoma

## Abstract

**Background and aims:**

As a result of increasing numbers of studies most recently, mitophagy plays a vital function in the genesis of cancer. However, research on the predictive potential and clinical importance of mitophagy-related genes (MRGs) in hepatocellular carcinoma (HCC) is currently lacking. This study aimed to uncover and analyze the mitophagy-related diagnostic biomarkers in HCC using machine learning (ML), as well as to investigate its biological role, immune infiltration, and clinical significance.

**Methods:**

In our research, by using Least absolute shrinkage and selection operator (LASSO) regression and support vector machine- (SVM-) recursive feature elimination (RFE) algorithm, six mitophagy genes (ATG12, CSNK2B, MTERF3, TOMM20, TOMM22, and TOMM40) were identified from twenty-nine mitophagy genes, next, the algorithm of non-negative matrix factorization (NMF) was used to separate the HCC patients into cluster A and B based on the six mitophagy genes. And there was evidence from multi-analysis that cluster A and B were associated with tumor immune microenvironment (TIME), clinicopathological features, and prognosis. After then, based on the DEGs (differentially expressed genes) between cluster A and cluster B, the prognostic model (riskScore) of mitophagy was constructed, including ten mitophagy-related genes (G6PD, KIF20A, SLC1A5, TPX2, ANXA10, TRNP1, ADH4, CYP2C9, CFHR3, and SPP1).

**Results:**

This study uncovered and analyzed the mitophagy-related diagnostic biomarkers in HCC using machine learning (ML), as well as to investigate its biological role, immune infiltration, and clinical significance. Based on the mitophagy-related diagnostic biomarkers, we constructed a prognostic model(riskScore). Furthermore, we discovered that the riskScore was associated with somatic mutation, TIME, chemotherapy efficacy, TACE and immunotherapy effectiveness in HCC patients.

**Conclusion:**

Mitophagy may play an important role in the development of HCC, and further research on this issue is necessary. Furthermore, the riskScore performed well as a standalone prognostic marker in terms of accuracy and stability. It can provide some guidance for the diagnosis and treatment of HCC patients.

## Introduction

HCC is the world’s fifth most prevalent malignancy, with the third highest fatality rate (1). Effective HCC therapy and diagnostic procedures continue to be significant concerns. Although there are several therapies for HCC, such as surgery, transcatheter arterial chemoembolization (TACE), and targeted therapies, the recurrence rate remains high. As a result, effective diagnostic and therapeutic procedures for HCC require rapid diagnosis and accurate intervention. Unfortunately, conventional indicators such as AFP did not perform well in terms of diagnostic effectiveness (2). Furthermore, AFP levels rise in various benign and malignant disorders, including different types of malignant tumors, chronic liver disease, pregnancy, and so on (3). Thus, it is vital to discover specific diagnostic markers and detect HCC at an early stage.

The intracellular mitochondria are responsible for transmitting several complex signals, including those associated with cell growth, energy consumption, cell differentiation, cell repair, and cell death (4). A change in the functional complexity of mitochondria is a critical element in tumor progression. To adapt to the tumor environment, cancer cells exhibit anaerobic glycolysis (often called the Warburg effect), abnormal mitochondrial quality control, altered cell REDOX status, reactive oxygen species (ROS) production, and apoptosis (5). When exposed to stressors like hypoxia, nutrient deficiency, inflammation, DNA damage, and the usage of mitochondrial uncouplers, mitochondria can generate ROS or apoptotic factors, resulting in cellular damage or apoptosis (6–8). In this situation, mitochondria can be cleaned by a process known as mitophagy, a kind of selective autophagy. Mitophagy has been shown to play a function in either promoting or restricting carcinogenesis (9–12). Furthermore, many studies have revealed that mitophagy is involved in immune function regulation (13). However, the role of mitophagy in the pathogenesis of HCC, as well as its importance in diagnosis and therapy, is currently unexplained. Therefore, a comprehensive analysis of the biological function, immune infiltration, and clinical significance of mitophagy can provide a new idea for the diagnosis and treatment of HCC.

Modern technologies, including ML algorithms, have been created to cope with the increasing volume and complexity of cancer and other multi-omics data (14, 15). A crucial area of artificial intelligence (AI) that is rapidly increasing is ML, which allows computer technology to learn from data processing and improve on its own to predict outcomes without explicit programming (16). The classification and identification of diagnostic cancer biomarkers using machine learning and traditional bioinformatics may considerably increase cancer biomarker identification accuracy and give new guidelines for cancer detection and therapy (14, 17).

We used an ML algorithm to select six diagnostic biomarkers among 29 mitophagy genes for future investigation. Next, a cluster of 113 HCC patients from the GEO database and 367 HCC patients from the TCGA database was formed based on the expression of six mitophagy genes. The variations between mitophagy-related patterns were examined using multi-omics analysis, which included clinical relevance, survival analysis, and TIME. We developed the prognostic model (riskScore), which was demonstrated to be an independent prognostic marker. The riskScore can predict the overall survival rate of HCC patients. Furthermore, we discovered a relationship between riskScore and somatic mutation, TIME, chemotherapy efficacy, TACE and immunotherapy effectiveness in HCC patients.

## Materials and methods

### Tissue samples and clinical information

The HCC and paracancerous tissues (n = 40 for both) were procured for patients in the Department of Hepatobiliary Pancreatic Surgery, Northern Jiangsu People’s Hospital, and surgical therapy was provided to forty patients with primary HCC. The goals of the Declaration of Helsinki were followed when conducting the current investigation. The people who donated the samples gave their written, informed consent.

### Original data acquisition and processing

All patients in this investigation had transcriptome and clinical data collected using the Gene Expression Omnibus (GEO) databases (https://www.ncbi.nlm.nih.gov/gds) and Cancer Genome Atlas (TCGA) (https://cancergenome.nih.gov/) platform. The GEO data come from the GSE76427 microarray data. We also handled the clinical data of patients. In the TCGA, GEO, and ICGC cohorts, we retrieved baseline information, survival information, and pathological information for analysis. The TCGA database patients serve as a training set for constructing the prognosis model. In contrast, the International Cancer Genome Consortium (ICGC) database patients (https://dcc.icgc.org/projects/LIRI-JP) serve as a validation set for establishing and validating the prognosis model. To assess TACE sensitivity, we employed the GSE104580 in the research. From GeneCards (https://pathcards.genecards.org/), the 29 mitophagy genes were retrieved.

### Algorithms for LASSO regression and SVM-RFE identify diagnostic biomarkers

LASSO is a regression-based method that reduces the risk of overfitting by minimizing regression coefficients (18). Hence, reducing redundancy and eliminating redundant genes from these studies (19). SVM is one of the best techniques for feature selection and the most used classifier for microarray data (20). SVM-RFE is a feature selection approach based on SVM (21). The SVM-RFE process chooses the best genes to specify the least amount of classification error while minimizing overfitting (20). As a result, two machine learning techniques are frequently employed to find biomarkers and develop models that are reliable and interpretable. We used the glmnet package to conduct the LASSO regression approach and the e1071 package to generate an SVM model in this investigation. To determine the value with the least cross-validation error as HCC feature markers, two ML approaches, LASSO regression, and SVM-RFE, were utilized.

### Single-cell RNA-seq analysis

Tumor Immune Single-cell Hub databases (TISCH databases, http://tisch.comp-genomics.org/) gathered information from GEO and Array Express to create a single-cell RNA-seq (scRNA-seq) atlas. By displaying gene expression across many data sets at the single-cell or cluster level, TISCH compares various patients, therapy and response groups, tissue origins, cell types, and even cancer sorts. In this work, we used TISCH datasets to investigate the TIME heterogeneity of six mitophagy genes at the single-cell level in GSE140228 (22) by comparing five patients.

For riskScore research, the Single-cell RNA-seq data are available in GEO database, reference chip of GSE166635 (23). Red blood cell percentage is less than 3%, each cell contains between 250 and 5000 genes, and there are less than 300,000 copies of all genes expressed. The names of the first ten genes that were simultaneously highly variable were tagged, and we chose 2000 genes with the highest variations and colored them red.

### Consensus clustering analysis for MRGs

To study the numerous mitophagy-related patterns, we utilized the R package “ConsensusClusterPlus” to perform consensus clustering. To create a consistent grouping, the algorithm repeats the deposition and combines the results. K = 2 was the ideal cluster value, according to the cophenetic coefficient, contour, and sample size. The R package “prcomp” and all the selected genes associated with mitophagy were used to construct the principal component analysis (PCA) score system.

### Gene set enrichment analysis (GSEA)

The non-parametric, unsupervised GSEA method may transform a segregated gene expression matrix into a characteristic expression matrix for a particular gene set. The “enrichplot,” DOSE,” and “clusterProfiler” R packages were used to implement the technique. We looked at the statistical changes in the modified expression matrix using the “limma” package.

### Evaluation of the TIME

To establish TIME’s characteristics, the percentages of immune cell types in each sample were determined using the MCP-counter approach. Furthermore, we examined the association between the number of immune cells and riskScore using the Wilcoxon rank-sum test. To investigate the immunological state, we performed an ssGSEA analysis. The superior algorithm (24) was used to assess the Tumor Immunological Dysfunction and Exclusion (TIDE) for modeling the mechanisms of distinct tumor immune evasion.

### Enrichment of functional analysis of differentially expressed genes

Using T statistics and p values (p < 0.001), the “limma” tool was used to assess changes in gene expression following NMF clustering to identify DEGs between two distinct phenotypes. Next, we examined DEGs between mitophagy-related patterns using the Metascape web-based platform’s enrichment of the Gene Ontology (GO) and KEGG pathways (25).

### Prognostic signature establishment and validation

The relevant prognostic genes, which consisted of ten DEGs (G6PD, KIF20A, SLC1A5, TPX2, ANXA10, TRNP1, ADH4, CYP2C9, CFHR3, and SPP1), were discovered by applying the LASSO regression to the prognosis-related DEGs in the model of univariate Cox regression. Finally, we got the riskScore formula:


$$f\left(x\right)=\sum^{}\left(exp\;Genei\times \;coeffient\;Genei\right)$$

The R function “surv cutpoint” was used to divide the TCGA cohort into high- and low-risk groups after determining the appropriate riskScore cutoff. We assessed the predicted reliability of predictive models using Kaplan-Meier analysis (package of “survival”) and the receiver operating characteristic (ROC) curve (package of “timeROC”). The area under the curve (AUC) was used to quantify the ROC curve. We employed identical analytic methodologies, riskScore algorithm, and cutoff value to verify the signature in the ICGC cohort.

### The assessment of the effectiveness of chemotherapy and targeted drug

Maeser et al. created the OncoPredict R package (26). To predict *in vivo* drug responses in cancer patients, OncoPredict compares the gene expression profiles of tissues and cancer cell lines from the Broad Institute Cancer Cell Line Encyclopedia Genomics of Drug Sensitivity in Cancer (GDSC; https://www.cancerrxgene.org/) and (CCLE; https://portals.broadinstitute.org/ccle legacy/home) based on the half-maximal inhibitory concentration (IC50) of drugs in cancer cell lines. Unpaired t-tests were used to compare the sensitivity of the drugs (between the high- and low-risk groups) in 198 medicines. The significance level was chosen at P-value < 0.05.

### Quantitative real-time PCR

TRIzol Reagent (Invitrogen Carlsbad, CA, USA) was used to extract total RNA for assay from 40 matched HCC tissues and paracancerous tissues following the manufacturer’s instructions. Each sample’s total RNA was converted into cDNA using the PrimeScriptTM RT reagent Kit from Takara Bio Inc. in Japan. Real-time PCR was carried out in a Rotor-Gene 3000 device using an SYBR-Green PCR kit from Takara in Osaka, Japan (Corbett Life Science, Sydney, Australia). **Supplementary Table 1** lists the primers that were utilized.

### Statistical analysis

The statistical analysis for this study was carried out using R (version 4.0.5) software. To validate a clear difference between the two groups, we used the paired samples t-test or the Wilcoxon rank-sum test. The Kruskal-Wallis test was done to see if there was a significant difference between more than two groups. Spearman’s correlation analysis was used to determine the relevant coefficients between immune checkpoint gene expression, TMB, and riskScore. To show the frequency of gene mutations, we used the “maftools” software to create waterfall charts and established a statistical difference criterion of P-value < 0.05.

## Result

### Identification of feature biomarkers of mitophagy by ML algorithm

**Figure 1:**
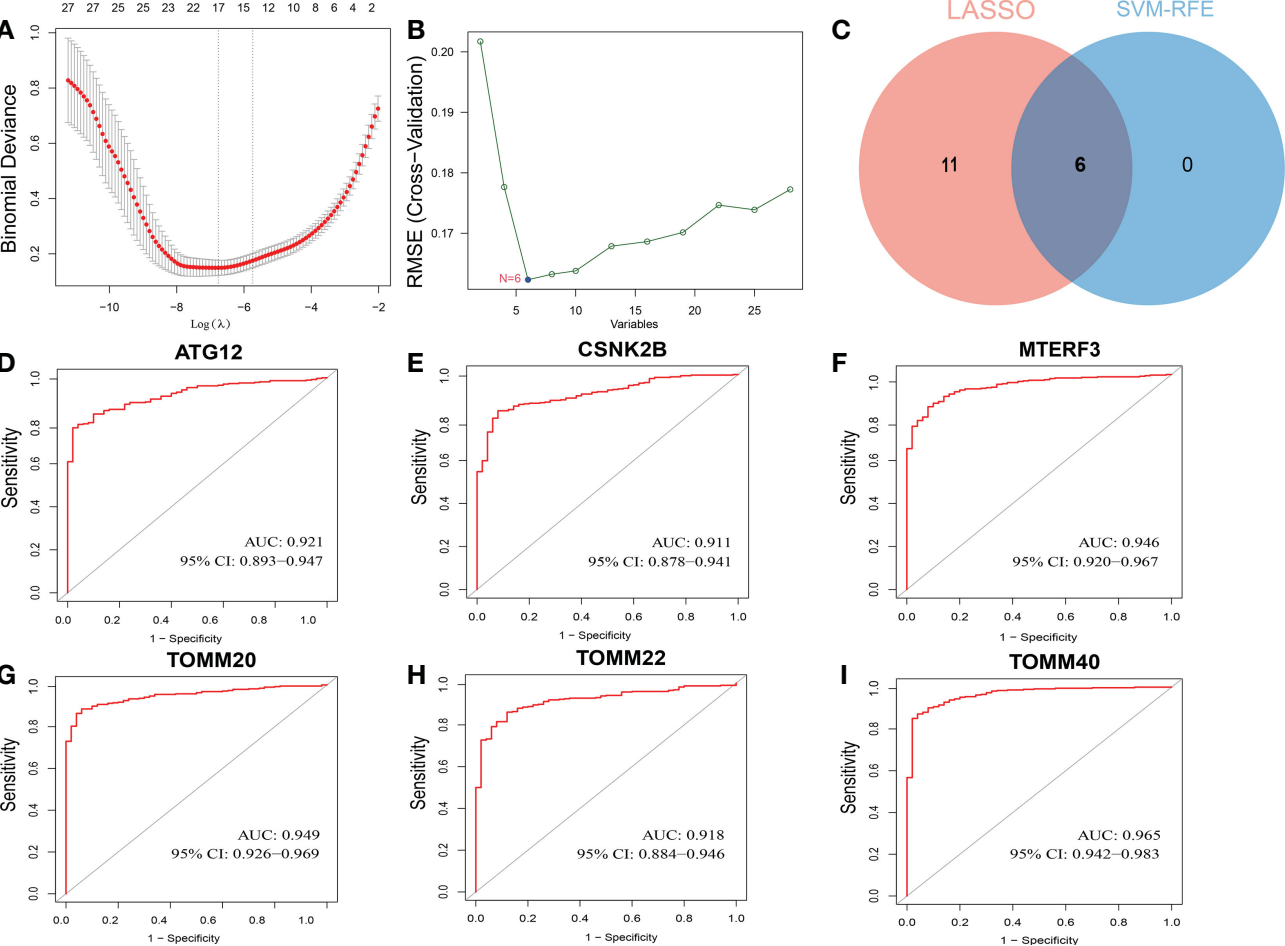
Screening process of diagnostic biomarker candidates for HCC diagnosis and several biomarkers were identified. **(A)** LASSO regression algorithm. **(B)** SVM-RFE algorithm. **(C)** Two ML algorithms take intersection to identify diagnostic feature genes. ROC curves of feature genes in experimental data set. **(D)** ATG12. **(E)** CSNK2B. **(F)** MTERF3. **(G)** TOMM20. **(H)** TOMM22. **(I)** TOMM40.

From the GeneCards, a total of twenty-nine mitophagy genes were obtained for this investigation. Then, to find putative HCC diagnostic biomarkers, we employed two different ML algorithm, LASSO regression and SVM-RFE, in the TCGA dataset. By narrowing the range of DEGs using the LASSO regression method, seventeen genes were identified as diagnostic indications of HCC (**Figure 1A**). And the SVM-RFE technique found six feature genes in DEGs (**Figure 1B**). The intersection of the two algorithms yielded six diagnostic feature genes (**Figure 1C**). According to **Figures 1D-I**, the AUC values of ATG12, CSNK2B, MTERF3, TOMM20, TOMM22, and TOMM40 were 0.921 (95% CI 0.893-0.947), 0.911 (95% CI 0.878-0.941), 0.946 (95% CI 0.920-0.912), 0.949 (95% CI 0.926-0.969), 0.918 (95% CI 0.884-0.946), and 0.965 (95% CI 0.942-0.983).

### The genetic variation landscape of six mitophagy gene regulators in HCC

**Figure 2:**
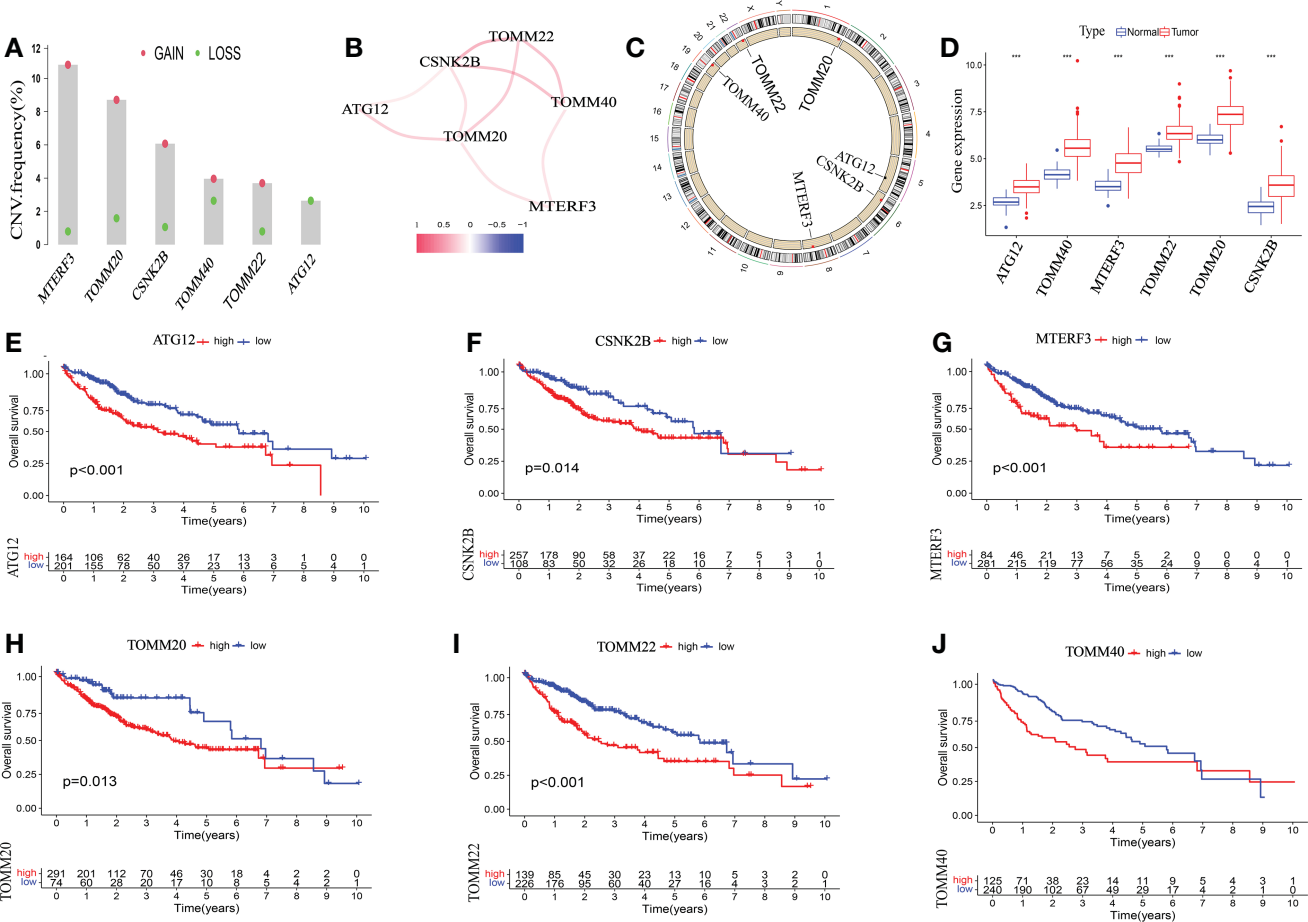
The genetic alterations of six mitophagy genes landscape in HCC. **(A)** The CNV alteration location of six mitophagy genes on chromosomes. **(B)** The CNV mutation frequency of six mitophagy genes was prevalent. The column represented the alteration frequency. The deletion frequency is red dot; The amplification frequency is blue dot. **(C)**The correlation among six mitophagy genes. **(D)** The differential expression levels in six mitophagy genes between normal and tumor samples. The asterisks represented the statistical P-value (***P < 0.001). **(E-J)** Kaplan–Meier analysis of six mitophagy genes between low and high expression.

Next, we investigated the involvement of six mitophagy genes in HCC. CNV (Copy number variations) mutations were found in six mitophagy genes studied. **Figure 2A** depicts the chromosomal locations of CNV changes in six mitophagy genes. The analysis of six mitophagy genes showed CNV mutations were prevalent. CSNK2B, MTERF3, TOMM20, TOMM22, and TOMM40 revealed widespread CNV amplification (**Figure 2B**). These genes were positively related to one another (**Figure 2C**). Further analysis demonstrated that six mitophagy genes significantly up-regulated in HCC samples (**Figure 2D**). Compared with high expression, Kaplan-Meier analysis (**Figures 2E-J**) showed substantially longer OS (Overall Survival) with low expression of six mitophagy genes in the TCGA dataset. These results proved the apparent connections and differences in the transcriptomic and genomic of six mitophagy genes landscape. Hence, expression and genetic variation in six mitophagy genes were critical in the course of HCC.

### Correlation between six mitophagy genes and the HCC immune microenvironment at the single-cell level

**Figure 3:**
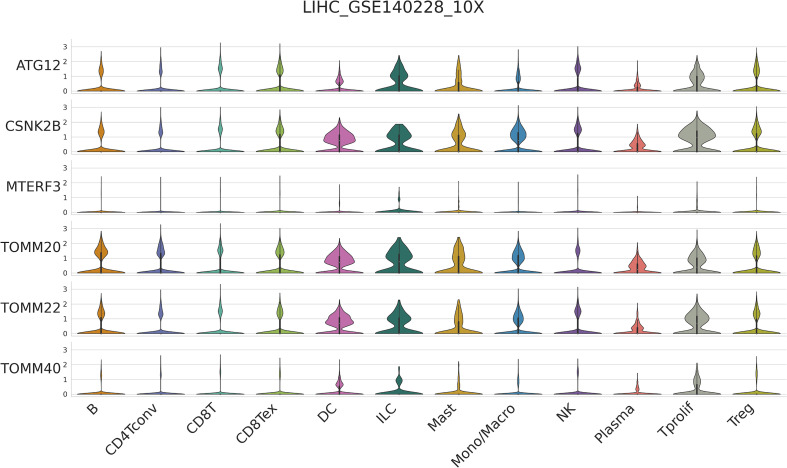
Single-cell RNA-seq analysis show the correlation between six mitophagy genes and the HCC Immune microenvironment by TISCH database.

It has been demonstrated that mitophagy affects immune cells’ infiltration of the tumor microenvironment (13). Thus, we investigated the expression of six mitophagy genes in the HCC TIME at the single-cell level using the TISCH database in LIHC_GSE140228. Violin Chart (**Figure 3**) shows that six mitophagy genes, specifically ATG12, CSNK2B, TOMM20, and TOMM22, were significantly expressed in immune cells, including B cells, CD4 T cells, CD8T cells, DC cells, ILC cells, mast cells, monocytes/macrophages, NK cells, plasma cells, Tprolif cells, and Treg cells. These results suggest that the expression of six mitophagy genes in HCC is closely related to immune cell infiltration.

### Consensus clustering of mitophagy-related patterns

**Figure 4:**
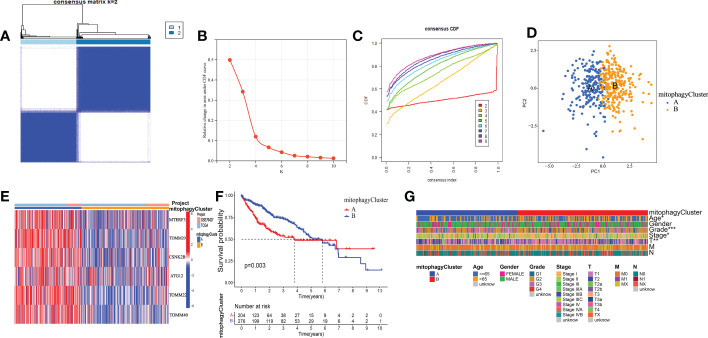
The prognostic value of the cluster A and cluster B. **(A)** Consensus matrix identified two clusters. **(B)** Delta area of the cluster analysis. **(C)** The cumulative distribution function of the cluster. **(D)** PCA indicated two components. **(E)** Transcription profile heatmap of six mitophagy genes in cluster A and cluster B. **(F)** Kaplan–Meier analysis of OS. **(G)** Clinical relevance of cluster A and cluster B in TCGA-HCC cohort. The asterisks represented the statistical P-value (*P < 0.05; **P < 0.01; ***P < 0.001).

We used the TCGA and GEO datasets to evaluate these six mitophagy genes to improve the analysis’s precision and reliability. We conducted consensus clustering on the mRNA expression patterns of six mitophagy genes in combined TCGA-HCC and GSE76427 samples. We found two molecular clusters (cluster A, cluster B, **Figures 4A-C**), and PCA revealed two distinct components (**Figure 4D**). We discovered that the transcription profile heatmaps of mitophagy genes differed considerably across clusters A and B. In cluster A, six mitophagy genes were highly elevated (**Figure 4E**). Furthermore, we looked at the clinical significance differences between the cluster A and B patterns. The OS of the cluster B pattern was significantly longer than that of the cluster A pattern, according to Kaplan-Meier analysis (**Figure 4F**). Finally, the chi-square test was employed to differentiate between the clinicopathological aspects of cluster A and B. The histologic grade, T stage, and pathologic stage distribution differed considerably between the cluster A and B patterns, as indicated in the figure (**Figure 4G**).

### TIME of mitophagy-related patterns

**Figure 5:**
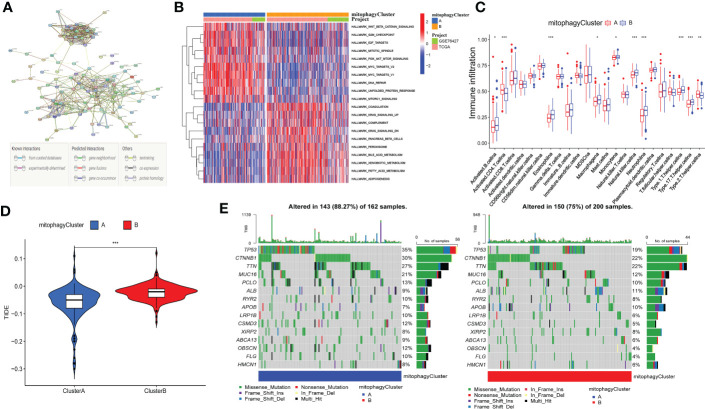
Correlation between mitophagy-related patterns and the TIME. **(A)** The protein-protein interaction network of DEGs. **(B)** Heatmap of GSVA analysis results. **(C)** The 23 immune cells shown in cluster A and cluster B with boxplots (*p < 0.05, **p < 0.01, ***p < 0.001). **(D)** The relative distribution of TIDE was compared between cluster A and cluster B. **(E)** The 30 genes with the highest mutation rate of cluster A and cluster B in Waterfall plots.

Additionally, we analyzed the DEGs between cluster A and cluster B. 226 differential genes were screened (**Figure S2**, **Supplementary Table 2**). These DEGs have multiple protein-protein interaction relationships (**Figure 5A**). We utilized GSVA (Gene set variation analysis) to research the biological molecular distinctions among the cluster A and cluster B patterns and the biological processes between these two distinct RNA processing patterns (**Figure 5B**). Wnt/-Catenin signaling, G2M checkpoint, E2F targets, PI3K/AKT mTOR signaling, MYC targets, DNA repair, unfolded protein response, and mTORC1 signaling was found to be considerably greater in cluster A. However, in the pathways of KRAS signaling, complement, Pancreas beta-cells, xenobiotic metabolism, peroxisome, fatty acid metabolism, bile acid metabolism, and adipogenesis, cluster B had a higher concentration. In addition, to contrast and demonstrate the interconnected richness of 23 immune infiltrating cell subpopulations between the cluster A and cluster B patterns, we used ssGSEA to construct a comparison graphic. (**Figure 5C**). We found that CD4 +T cell and T helper cell 2 were enriched in the cluster A. However, B cell, Eosinophil, Macrophage, monocyte, natural killer cell, neutrophil, T helper cell 1, and T helper cell 17 were enriched in the cluster B. In summary, cluster B appears to be infiltrated by more types of immune cells. One method of predicting the immunological escape of tumor cells is the TIDE score. Hence, a lower rate of immune checkpoint inhibitor (ICI) therapeutic response is indicated by a higher TIDE score. Surprisingly, the TIDE of cluster B was significantly higher than cluster A (**Figure 5D**). As indicated by the waterfall plots (**Figure 5E**), the highest mutation rate of ten genes in cluster A are TP53 (35%), TTN (30%), MUC16 (27%), CTNNBI (21%), PCLO (13%), ALB (9%), RYR2 (10%), APOB (7%), LRP1B (10%), and CSMD3 (12%). By contrast, the ten genes with the most significant rate of mutation in C2 are TP53 (19%), TTN (22%), MUC16 (22%), CTNNBI (12%), PCLO (10%), ALB (11%), RYR2 (8%), APOB (10%), LRP1B (6%), and CSMD3 (5%). In summary, we explored the function of cluster A and cluster B in biological role, immune infiltration and immunotherapy may have differences.

### The mitophagy-related signature establishment in TCGA-HCC cohort

**Figure 6:**
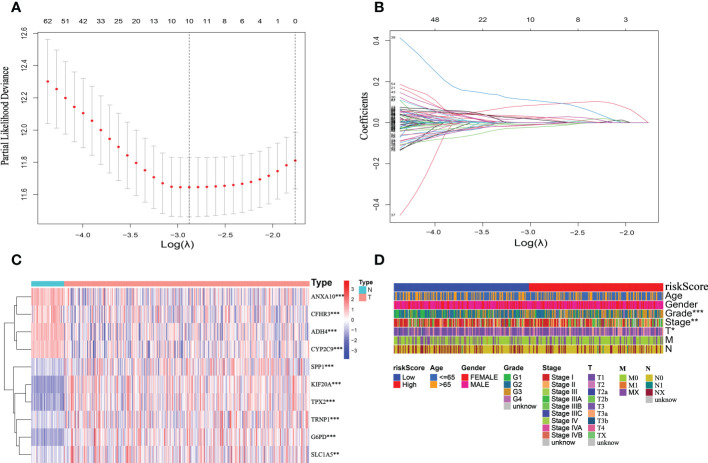
Establishment of the mitophagy-related signature according to the training set. **(A, B)** LASSO COX regression analysis. **(C)** The expression profile heatmap of ten genes (*p < 0.05, **p < 0.01, ***p < 0.001). **(D)** Clinical relevance of high-risk and low-risk groups in TCGA cohort.

Based on previous studies using DEGs in cluster A and cluster B, the results of univariate cox regression analysis showed that 145 genes were related to the prognosis of HCC (**Supplementary Table 3**). To extract the coefficient and ten mitophagy-related genes, LASSO Cox regression was then used to process the univariate Cox regression model, including G6PD, KIF20A, SLC1A5, TPX2, ANXA10, TRNP1, ADH4, CYP2C9, CFHR3, and SPP1, were selected according to the bare minimum need (**Figures 6A, B**). As shown in the heat map (**Figure 6C**), compared with normal tissues, G6PD, KIF20A, SLC1A5, TPX2, TRNP1 and SPP1 are significantly up-regulated in HCC, while ANXA10, ADH4, CYP2C9 and CFHR3 are significantly down-regulated. The quantitative indicator was calculated as follows: riskScore = (0.083347 × G6PD expression) + (0.11351 × KIF20A expression) + (0.02069 × SLC1A5 expression) + (0.02567 × TPX2 expression) + (-0.01388 × ANXA10 expression) + (0.02062 × TRNP1 expression) + (-0.00805 × ADH4 expression) + (-0.01140 × CYP2C9 expression) + (-0.02398 × CFHR3 expression) + (0.03213 × SPP1 expression). Following that, we estimated riskScore for each patient using the following formula. The patients were separated into two groups according to the appropriate cutoff value for riskScore (cut point = 0.7713): high-risk (n = 182) and low-risk (n = 183). We also investigated the significance of the riskScore and clinicopathological features. Histologic grade, T stage, and pathologic stage differ between low- and high-risk groups statistically significantly in the TCGA cohort (**Figure 6D**).

### The validation of mitophagy-related signatures in the ICGC-HCC dataset

**Figure 7:**
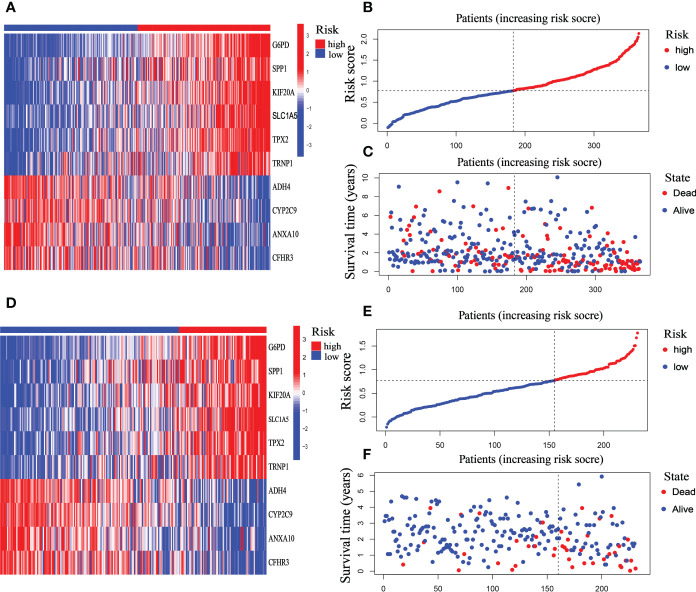
Prognostic characteristic of the ten-gene signature model in the TCGA and ICGC cohort. **(A)** The expression profile heatmap of ten mitophagy-related genes in TCGA dataset. **(B)** riskScore distribution in TCGA dataset. **(C)** Survival status heatmap in TCGA dataset. **(D)** The expression profile heatmap of ten mitophagy-related genes in ICGC dataset. **(E)** riskScore distribution in ICGC dataset. **(F)** Survival status heatmap in ICGC dataset.

The riskScore model was tested on the ICGC dataset, which comprised 231 HCC patients, to validate the precision and stability of our findings. The TCGA set was separated into two groups utilizing the identical cutoff value (cut point = 0.7713): high-risk (n = 76) and low-risk (n = 155). And as the figure exhibition, the trend of the TCGA-HCC dataset and the ICGC-HCC dataset were similar in the heatmaps of ten mitophagy-related genes expression profiles, the riskScore distribution, and the status of survival (**Figures 7A-F**).

### Clinical relevance of the mitophagy-related signature in TCGA dataset

**Figure 8:**
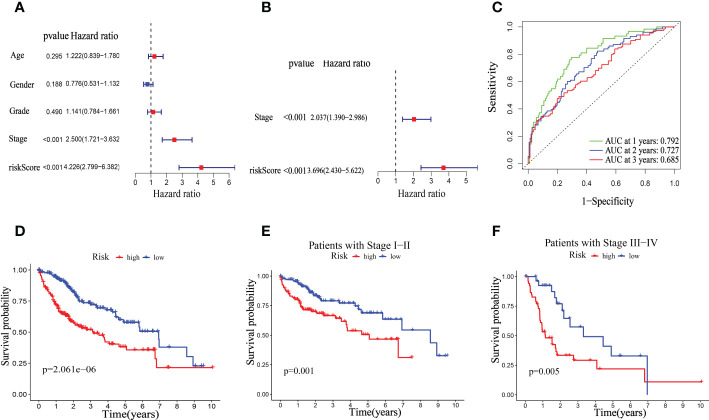
Univariate and multivariate Cox regression analyses for OS in the TCGA train group. Univariate **(A)** and multivariate **(B)** Cox regression analysis of riskScore and clinicopathological parameters in TCGA dataset. **(C)** Time-dependent ROC curve of riskScore. **(D)** Kaplan–Meier analysis between riskScore-defined groups. **(E)** Kaplan–Meier analysis between riskScore-defined groups in patients with stage I-II. **(F)** Kaplan–Meier analysis between riskScore-defined groups in patients with stage III-IV.

We investigated the significance of the riskScore and clinicopathological variables to further examine the mitophagy-related hallmark clinical advantages. Using univariate Cox regression analysis, we discovered that riskScore and pathological stage were hazard variables in the TCGA dataset (**Figure 8A**). We demonstrated the riskScore was an effective independent prognostic factor in the TCGA-HCC dataset using multivariate Cox regression (hazard ratio (HR) = 3.696 (2.430–5.622), p < 0.001, **Figure 8B**). **Figure 8C** shows that the 1-, 2-, and 3-year OS survival rates of AUC were, respectively, 0.792, 0.727, and 0.685. Kaplan-Meier analysis revealed that patients in the low-risk group had considerably longer OS than those in the high-risk group (**Figure 8D**). And in both the early and advanced stages, the low-risk group’s OS was much longer than the high-risk group’s. (**Figures 8E, F**).

### Clinical relevance of the mitophagy-related signature in ICGC dataset

**Figure 9:**
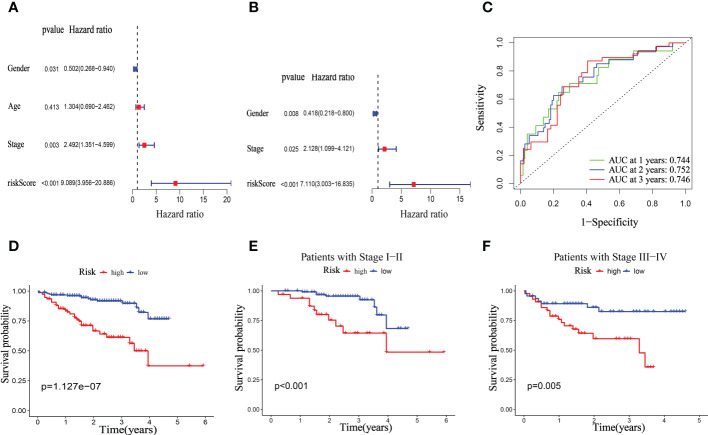
Univariate and multivariate Cox regression analyses for OS in ICGC test group. Univariate **(A)** and multivariate **(B)** Cox regression analysis of riskScore and clinicopathological parameters in ICGC dataset. **(C)** Time-dependent ROC curve of riskScore. **(D)** Kaplan–Meier analysis between riskScore-defined groups. **(E)** Kaplan–Meier analysis between riskScore-defined groups in patients with stage I-II. **(F)** Kaplan–Meier analysis between riskScore-defined groups in patients with stage III-IV.

The clinical significance of the mitophagy-related signal in the ICGC dataset was then confirmed. Similar to the TCGA dataset, similar findings were achieved. The univariate Cox regression analysis revealed that the pathological stage and riskScore were risk factors (**Figure 9A**). Using multivariate Cox regression, it was discovered that riskScore was an independent prognostic predictor in the ICGC dataset hazard ratio (HR) = 7.110 (3.003–16.835), *p* < 0.001, **Figure 9B**). In the ICGC dataset, the 1-, 2-, and 3-year OS survival rates of AUC were, respectively, 0.744, 0.752, and 0.746 (**Figure 9C**). The TCGA database shows that patients in the high-risk category had considerably worse overall survival (OS) than those in the low-risk group (**Figure 9D**). Furthermore, in both the early and later advanced stages, the OS of the low-risk group was observably longer than that of the high-risk group. (**Figures 9E, F**).

### Correlation between the time and mitophagy-related signature

**Figure 10:**
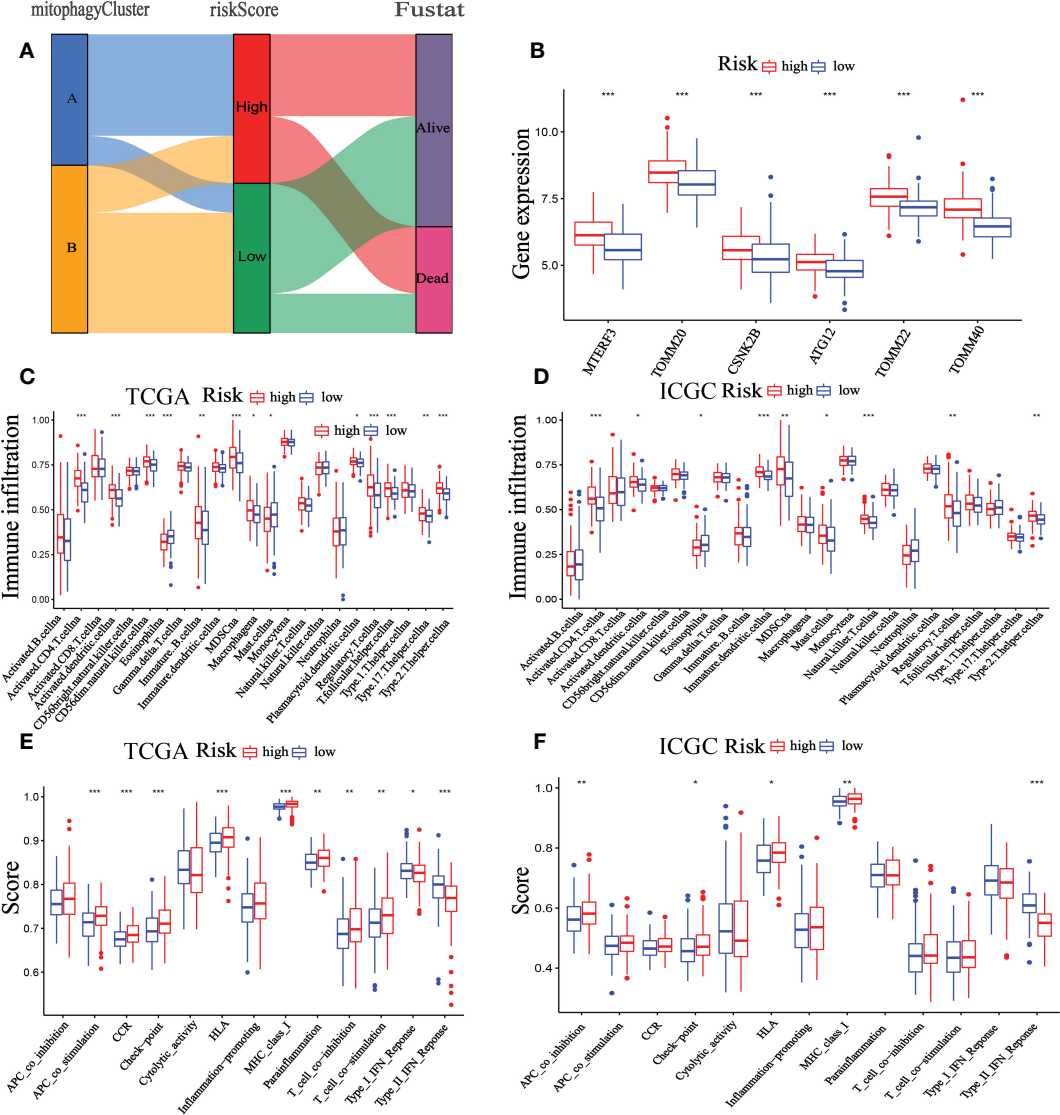
Correlation between the TIME and mitophagy signature. **(A)** The Sankey diagram shows the distribution of high-risk groups and low-risk groups in cluster A and cluster B patterns. **(B)** Expression of the mitophagy-related gene in the low-risk group and high-risk group. The ssGSEA results of different risk groups in the TCGA cohort **(C, E)** and ICGC cohort **(D, F)**. The 23 immune cell scores **(C, D)** and 13 immune-related functions **(E, F)** were shown in boxplots. *p < 0.05; **p < 0.01; ***p < 0.001.

Existing research showed that during carcinogenesis, mitophagy activates an adaptive immune response (27). Thus, we detected the association between the TIME and the mitophagy-related signature. We examined the distribution of low- and high-risk groups in the TCGA cohort using the cluster A and B patterns. The results revealed that the high-risk groups are nearly entirely concentrated in Cluster A, whereas Cluster B is primarily made up of low-risk groups (**Figure 10A**). Furthermore, six mitophagy genes expression were evaluated in low- and high-risk groups. The findings reveal that the mitophagy genes (ATG12, CSNK2B, MTERF3, TOMM20, TOMM22, and TOMM40) are more expressed in the high-risk group compared to the low-risk group (**Figure 10B**). The previous research showed that mitophagy-related clusters may be related to immune function and immunotherapy, and we had done corresponding research on the immune function of mitophagy-related signatures. Furthermore, Immune cells such as NK cells, CD4 + T cells, CD8 + T cells, and others serve as the biological basis of immunotherapy (28). To examine the relationship between immunological state and riskScore, we generated enrichment scores for several immune cell subpopulations using ssGSEA. According to the findings, both the TCGA and the ICGC dataset showed that patients with high-risk scores had higher levels of CD4 + T cells, activated dendritic cells, MDSC, Mast cell, NK cells, regulatory T cell, and T helper cell 2 infiltrations (**Figures 10C, D**). The ssGSEA results from the TCGA-HCC and ICGC-HCC datasets showed that the high-risk group enriched for the majority of immune-related activities. (**Figures 10E, F**).

### HCC single-cell subpopulations

**Figure 11:**
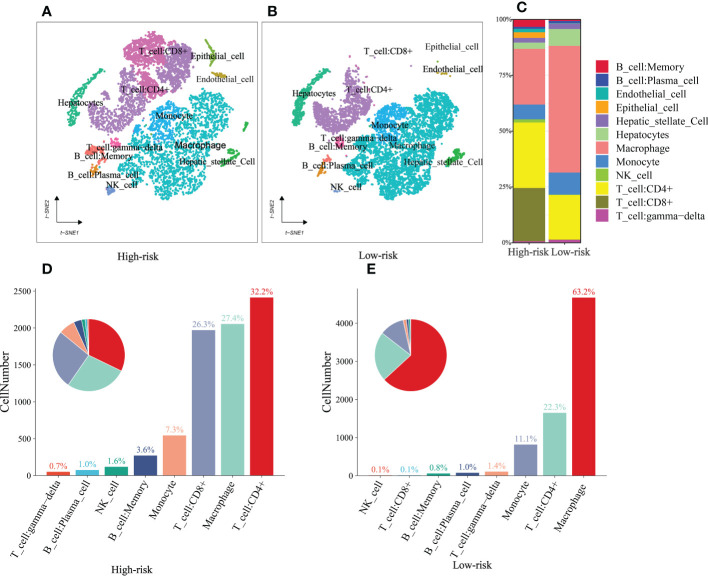
single-cell sequencing **(A, B)** t‐SNE plot of 16475 cells showing eight major cell types in high- and low group. **(C)** The distribution of Memory B cell, Plasma cell, Endothelial cell, Epithelial cell, Hepatic stellate Cell, Hepatocytes, Macrophage, Monocyte, NK cell, CD4+T cell, CD8+T cell, and γδT cells in the low- and high-risk groups. **(D, E)** The ratio of immune cells in the high- and low-risk groups.

Single-cell sequencing was used to verify the previous findings. Following this thorough quality check, we received 16475 single cells. Following dimension reduction by PCA, we discovered that the cells were arranged into twenty clusters (**Figure Supplements 2**). The top ten genes in each cluster had considerably greater expression than the other groups. We categorized these clusters into Memory B cell, Plasma cell, Endothelial cell, Epithelial cell, Hepatic stellate Cell, Hepatocytes, Macrophage, Monocyte, NK cell, CD4+T cell, CD8+T cell, and γδT cells (**Figures 11A, B**). Consistent with the previous analysis, the high-risk group had a higher proportion of immune cells, such as B cells, NK cells, CD4+T cells, and CD8+T cells, while the low-risk group had higher Macrophage, Hepatocyte and γδT cells than those in the high-risk group (**Figure 11C**). In addition, we further analyzed the differences between the components of immune cells in the high- and low-risk group (**Figures 11D, E**). As shown in the figure, the ratio of immune cell composition differs significantly between the two groups. The first three cells are CD4+T cells (32.2%), Macrophage (27.4%) and CD8+T cells (26.3%) in the high-risk group, while, Macrophage (63.2%), CD4+T cells (22.3%) and Monocyte (11.1%) in the low-risk group.

### The connection between Mitophagy-Related signatures and somatic mutation

**Figure 12:**
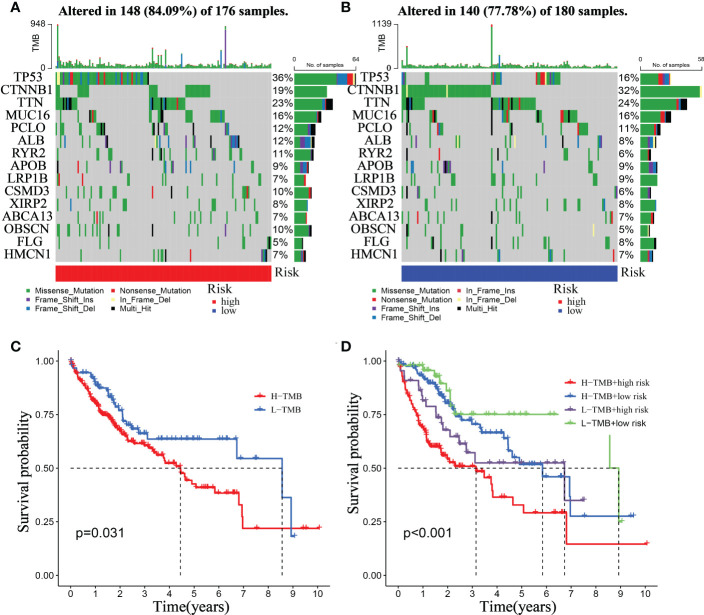
Relationship between the mitophagy-Related Signature and somatic mutation. Waterfall plots of 30 genes with the highest mutation rate in the high-risk group **(A)** and the low-risk group **(B)**. **(C)** Kaplan–Meier analysis of TMB in HCC patients. **(D)** Kaplan–Meier analysis of the correlation between riskScore and TMB.

Tumor formation is frequently triggered by the accumulation of mutations (29). As a result, we looked at the distinction between high- and low-risk somatic mutations (**Figure 12A**). In the high-risk group, the top five genes with the most significant mutation rates were TP53 (36%), CTNNB1 (19%), TTN (23%), MUC16 (16%), and PCLO (12%) (**Figure 12B**). According to the data, the low-risk group exhibits higher immune-related alterations. The patients were divided into high- and low- TMB groups based on the suitable TMB threshold. And the findings revealed that the greater the TMB value of HCC patients, the shorter their life expectancy (**Figure 12C**, p = 0.031). Based on RiskScore and the appropriate TMB cutoff value, patients in the TCGA were split into four categories: low-TMB+ low-risk, low-TMB+ high-risk, high-TMB+ high-risk, and high-TMB+ low-risk. The low-TMB+ low-risk group lived significantly longer than the high-TMB+ high-risk group (**Figure 12D**, p <0.001).

### Sensitivity of mitophagy-related model to chemotherapy, TACE and immunotherapy

**Figure 13:**
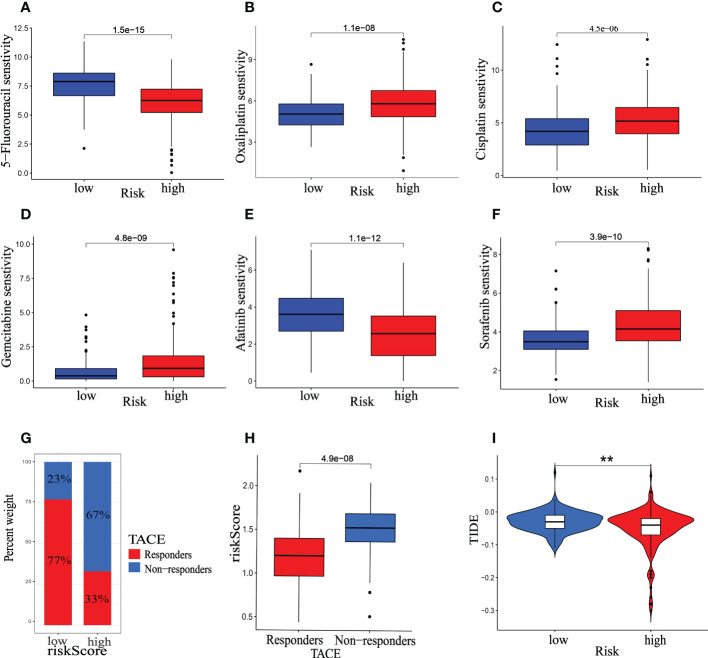
Therapeutic benefit of the riskScore. **(A–F)** Correlation between the mitophagy-related Signature and IC50 values of chemotherapy and targeted drugs, including **(A)** 5-Fluorouracil, **(B)** Oxaliplatin, **(C)** Cisplatin, **(D)** Gemcitabine, **(E)** Afatinib, **(F)** Sorafenib. **(G)** The distribution of TACE- response group versus TACE- None-response group in the low- and high-risk groups, and **(H)** the relative distribution of riskScore in TACE- response group versus TACE- None-response group. **(I)** The relative distribution of TIDE was compared between the low- and high-risk groups. The asterisks represented the statistical P-value (*p < 0.05, **p < 0.01, ***p < 0.001).

In the treating advanced HCC, Chemotherapy, TACE and immunotherapy have demonstrated therapeutic benefits, improving patients’ OS and progression-free survival. Nonetheless, adverse medication responses and resistance remain substantial barriers to developing pharmacological treatment (30). Additionally, further studying the differences between patients in various risk categories under our prognostic model type, we evaluated gene expression in patients with high- and low-risk scores and identified 1471 DEGs. DEGs were used to analyze GO and KEGG enrichment (**Figure S4**). According to the analyses of GO and KEGG enrichment, drug metabolism pathway is a significant part of DEGs enrichment function. Therefore, the relationship between riskScore and the IC50 values of targeted drugs and chemotherapy was calculated by the package of “OncoPredict” in R. Our findings demonstrate the IC50 values of Cisplatin, Gemcitabine, Oxaliplatin, and Sorafenib were higher in the high-risk group than in the low-risk group, whereas 5-Fluorouracil and Afatinib were lower in the high-risk group. (**Figures 13A-F**). Furthermore, the therapeutic response to TACE was examined between groups at low and high risk. The GSE104580 TACE chip was divided into the TACE-response and the TACE-Non-response group. In the low-risk group, 23% of patients did not respond to TACE, whereas 67% of those in the high-risk group responded (**Figure 13G**). And the proportion of patients responding to TACE was significantly lower in the high-risk score group than in the low-risk group (**Figure 13H**). Our analysis also found that the TIDE score was lower in the high-risk group (**Figure 13I**, P<0.01), indicating that patients in the high-risk category may be more susceptible to immunotherapy.

### Quantitative real-time polymerase chain reaction (qRT-PCR)

**Figure 14:**
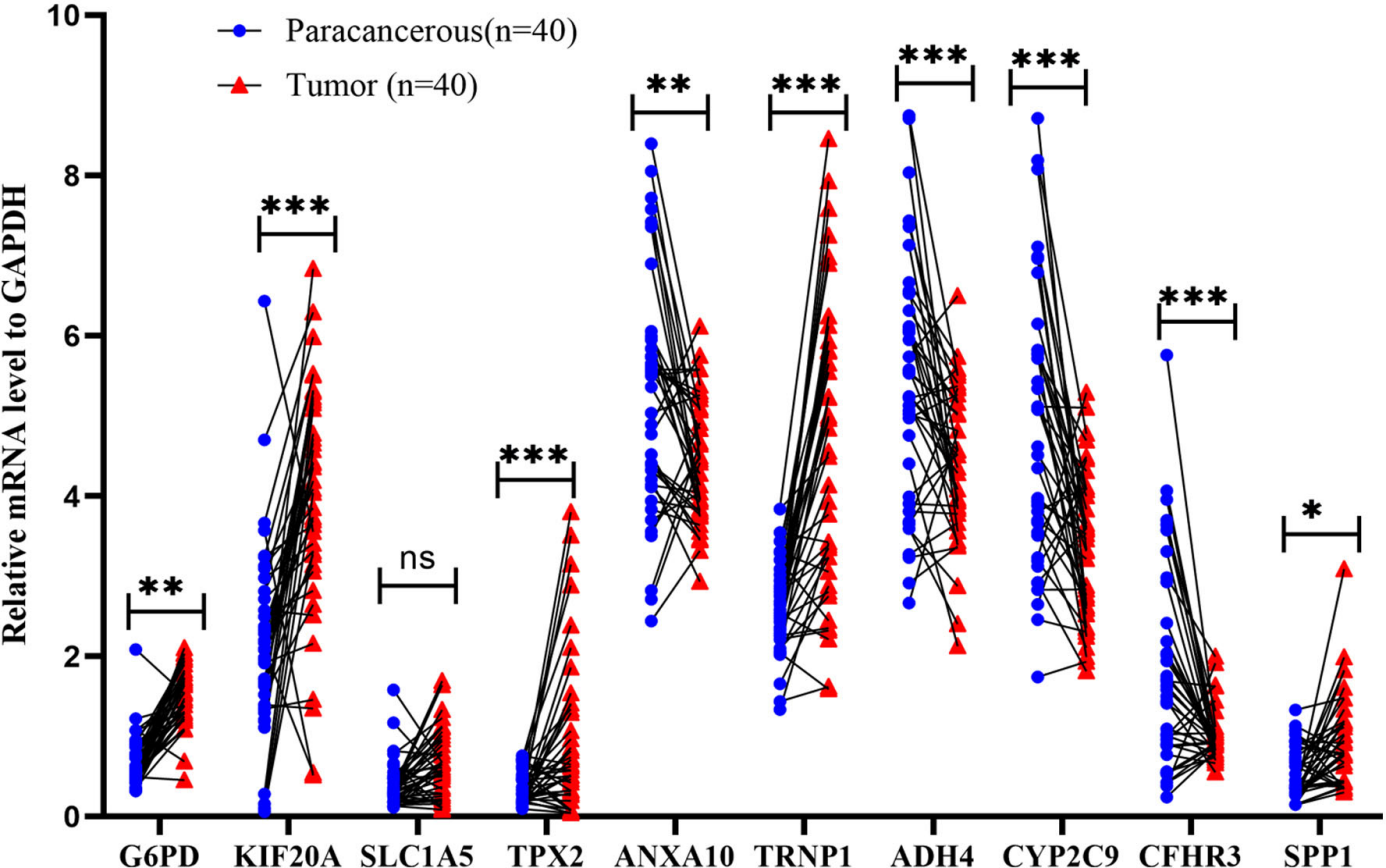
Validation of mRNA expression by real-time PCR. mRNA expression of ten genes related to mitophagy in 40 HCC tissues and paracancerous tissues; ns: not statistically significant, **: p < 0.01, ***: p < 0.001.

To corroborate the expression of risk genes in HCC, qRT-PCR was used to determine the expression of genes related to mitophagy (G6PD, KIF20A, SLC1A5, TPX2, ANXA10, TRNP1, ADH4, CYP2C9, CFHR3, and SPP1). In 40 HCC and paracancerous tissues, the results showed that the relative mRNA expression of G6PD, KIF20A, TPX2, TRNP1, and SPP1 was higher in HCC tissues. In comparison, the relative mRNA expression of ANXA10, ADH4, CYP2C9, and CFHR3 was higher in paracancerous tissues (**Figure 14**). These results suggested that it is reasonable to group according to the risk score.

## Discussion

In this study, we identified six mitophagy genes (ATG12, CSNK2B, MTERF3, TOMM20, TOMM22, and TOMM40) as diagnostic feature biomarkers for HCC according to a combination of ML algorithms and classical bioinformatics. Existing research shows that during carcinogenesis, mitophagy activates an adaptive immune response (27). And our research found that the expression of six mitophagy genes in HCC is closely related to clinical prognosis and immune cell infiltration. Next, we combined TCGA-HCC and GSE76427, we performed the consensus clusters and identified two molecular clusters (cluster A and cluster B). Compared to the cluster A pattern, the cluster B pattern indicated a longer OS. Furthermore, In the cluster A pattern, the proportion of advanced HCC patients had higher T stages. These two mitophagy-related patterns also displayed enriched immune cell infiltration and distinct biological pathways. We discovered that the cluster B appears to be infiltrated by more types of immune cells in the TCGA cohort. However, we observed that the TIDE of the cluster B pattern was higher than cluster A pattern. The results confirmed that mitophagy genes play an essential role in modulating the immunological landscape of HCC.

The DEGs between cluster A and cluster B patterns were then investigated. Using LASSO regression, we built a prognostic model in the TCGA-HCC cohort, which included ten mitophagy-related genes (G6PD, KIF20A, SLC1A5, TPX2, ANXA10, TRNP1, ADH4, CYP2C9, CFHR3, and SPP1). We demonstrated that the model was applicable in clinical. HCC patients were split into high- and low-risk groups based on their riskScore, and several analyses were done. Patients in the high-risk group had a lower OS than those in the low-risk group, demonstrating that the riskScore is associated with tumor progression or poor prognostic events. It was also shown in another external ICGC-HCC cohort. We found that the riskScore was a valuable independent prognostic factor in both the TCGA-HCC and the ICGC-HCC cohorts using univariate and multivariate Cox regression.

Since riskScore was constructed based on mitophagy genes, and as mentioned earlier, mitophagy genes were closely related to immune cell infiltration, we further explored the relationship between riskScore and immune cell infiltration and validated it by single cell sequencing. Interestingly, patients in high-risk group exhibited greater levels of infiltration of immune cells than low-risk group. What’s more, we further analyzed the differences between the components of immune cells in the high- and low-risk group. And, the ratio of immune cell composition differs significantly between the two groups. However, immune cells are widely established to be directly associated with tumor growth (31–35) or anti-tumor treatment (36–39). Therefore, comprehensive analysis of the above, we presume that riskScore may influence patient prognosis *via* the TIME.

Cancer patients with increased TMB had a higher likelihood of long-lasting and efficient treatment responses (40). The low-risk group had a lower TMB than the high-risk group, according to our data. In contrast, patients in the low-risk group showed more immune-related changes than those in the high-risk group. This is consistent with the results on immune infiltration presented above.

For advanced HCC, chemotherapy, immunocompetent individuals (ICIs), and TACE are all viable therapeutic options. In this investigation, we confirmed that the IC50 values of Cisplatin, Gemcitabine, Oxaliplatin, and Sorafenib were lower in the low-risk group than in the high-risk group, whereas 5-Fluorouracil and Afatinib were lower in the high-risk group. The previous analysis showed riskScore is a useful indicator for determining immunological status. As a result, we intended to investigate riskScore’s response rate to ICI treatment based on the TIDE score further. Interestingly, the low-risk group had a higher TIDE score, which suggests that they may respond worse to ICI therapy. In addition, we find that TACE treatment regimens may be more beneficial for low-risk patients. According to the analysis above, including immunotherapy, chemotherapy, and TACE, riskScore has significant guiding relevance for HCC treatment.

Collectively, we uncovered the mitophagy-related diagnostic biomarkers in HCC using ML algorithms and found they are closely related to biological function, immune infiltration, clinical prognosis. This suggests that mitophagy may play an important role in the development of HCC, and further research on this issue is necessary. Furthermore, we constructed a reliable prognostic model (riskScore). The phenotype of HCC patients may be quantified and individualized using riskScore by performing a complete review of the cellular, molecular, and clinical aspects of HCC patients. RiskScore is an important independent prognostic measure for choosing chemotherapy, TACE treatments and immunotherapy for HCC patients. Yet, our analysis includes limitations that need to be addressed further. Although the 29 genes in the mitophagy pathway we obtained from GeneCards can reflect the important role of mitophagy in HCC through analysis, based on the complexity of mitophagy function and the diversity of genetic phenotypes, these 29 genes cannot fully reflect the significance of mitophagy in HCC, and further experimental research is required to deepen our understanding of the link between mitophagy and HCC. Moreover, multicenter, high sample size, and prospective investigations, mainly as retrospective research, are necessary to corroborate the reliability of our constructed prognostic model.

## Data availability statement

The original contributions presented in the study are included in the article/**Supplementary Material**. Further inquiries can be directed to the corresponding authors.

## Ethics statement

The studies involving human participants were reviewed and approved by Ethics Committee of Northern Jiangsu People’s Hospital. The patients/participants provided their written informed consent to participate in this study.

## Author contributions

D-YT, JC, JZ, D-SB, and RP designed and implemented the research. G-QJ and S-JJ collated and analyzed the data. D-YT, JC, JZ, D-SB, and RP provided technical support. RP and JZ provided the language polishing for this article. D-YT wrote the manuscript. D-SB and JC revised the manuscript. All authors contributed to the article and approved the submitted version.

## Supplementary material

Supplementary Figure 1 Flow chart of our study.

Supplementary Figure 2 The heatmap of DEGs between cluster A and cluster B patterns.

Supplementary Figure 3 Quality control of single-cell sequencing data. **(A)** Gene expression levels in each cell of the 2 samples were in the range of 250-5000, and the percentage of mitochondrial, ribosome, and hemoglobin genes was controlled below 15%. **(B)** We chose 2000 hypervariable genes from all of the genes, which are in red, and tagged the first ten. **(C)** Reduced-dimensional representations of the cells are visualized in the scatter plot. **(D)** After dimension reduction through PCA, we found that the cells were clustered into 20 clusters.

Supplementary Figure 4 Enrichment analysis of differentially expressed genes in the high- and low-risk group in the TCGA cohort. **(A, B)** In GO and KEGG analyses we found that these genes were mainly associated with drug metabolism pathway.
