# Supplementary figures and images for: Identification of the mitophagy-related diagnostic biomarkers in hepatocellular carcinoma based on machine learning algorithm and construction of prognostic model
Source: Front Oncol. 2023 Mar 1;13:1132559. doi: 10.3389/fonc.2023.1132559 (PMC10014545; doi:10.3389/fonc.2023.1132559)

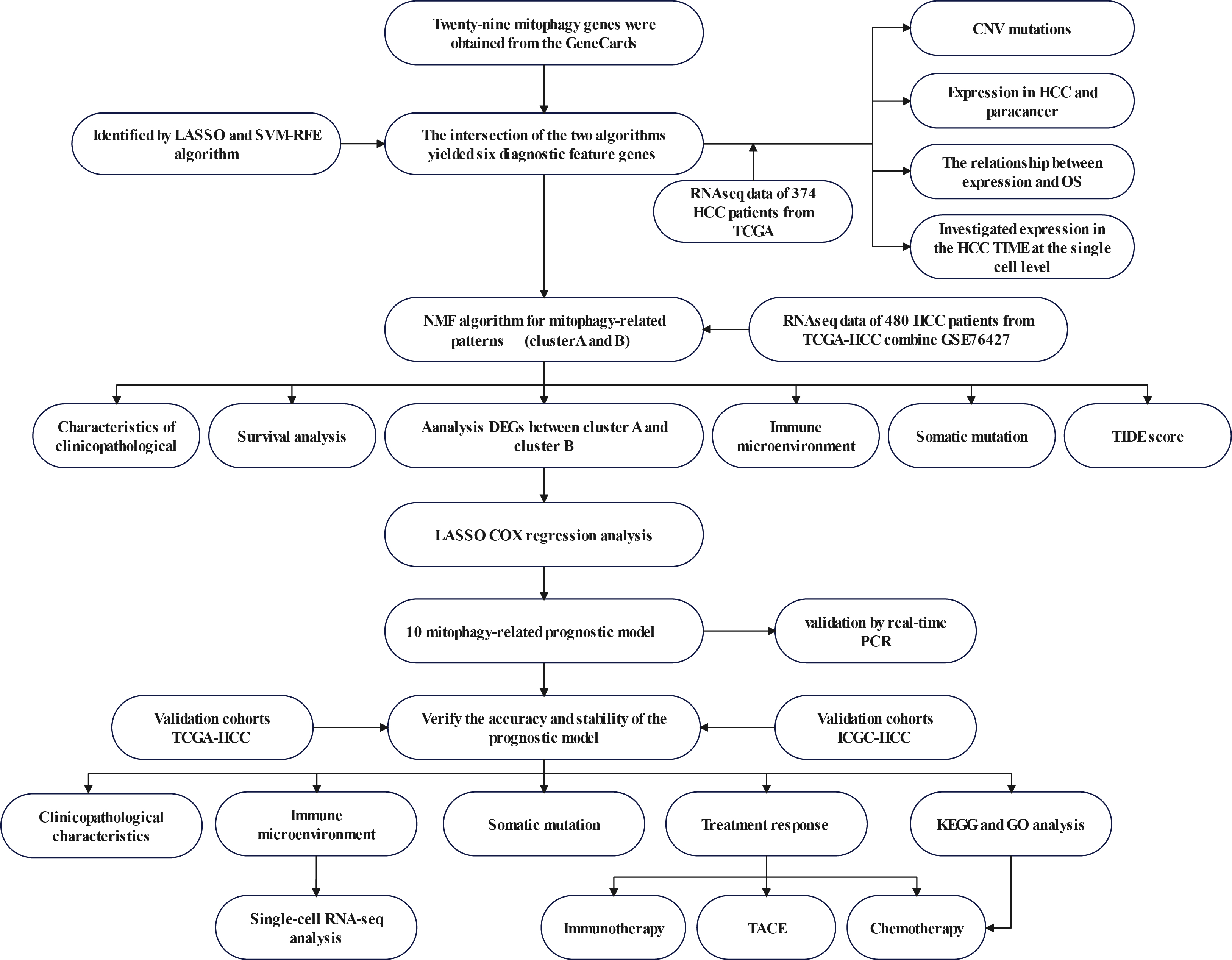

Supplement: Supplementary Figure 1 — Flow chart of our study. [file Image_1.jpeg]

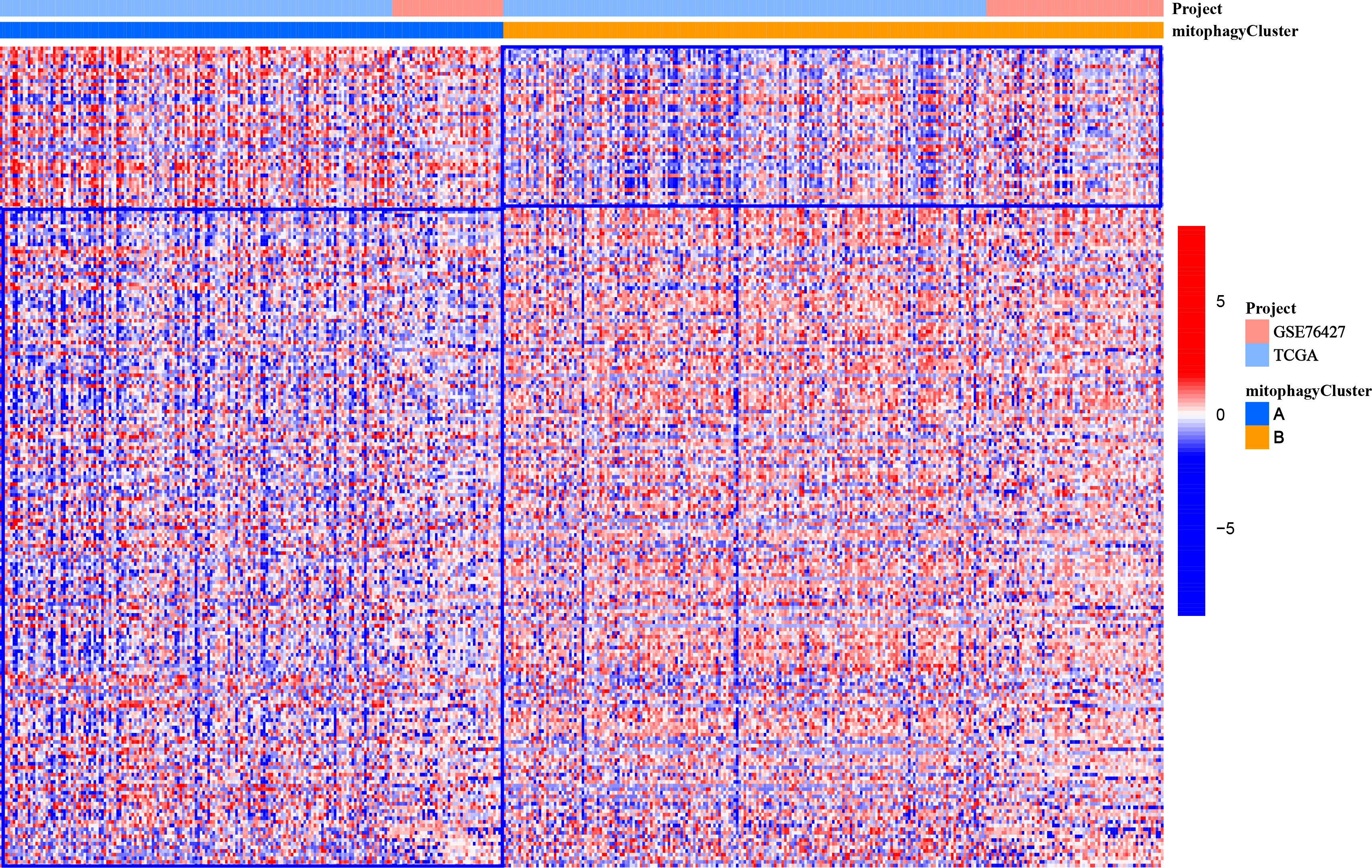

Supplement: Supplementary Figure 2 — The heatmap of DEGs between cluster A and cluster B patterns. [file Image_2.jpeg]

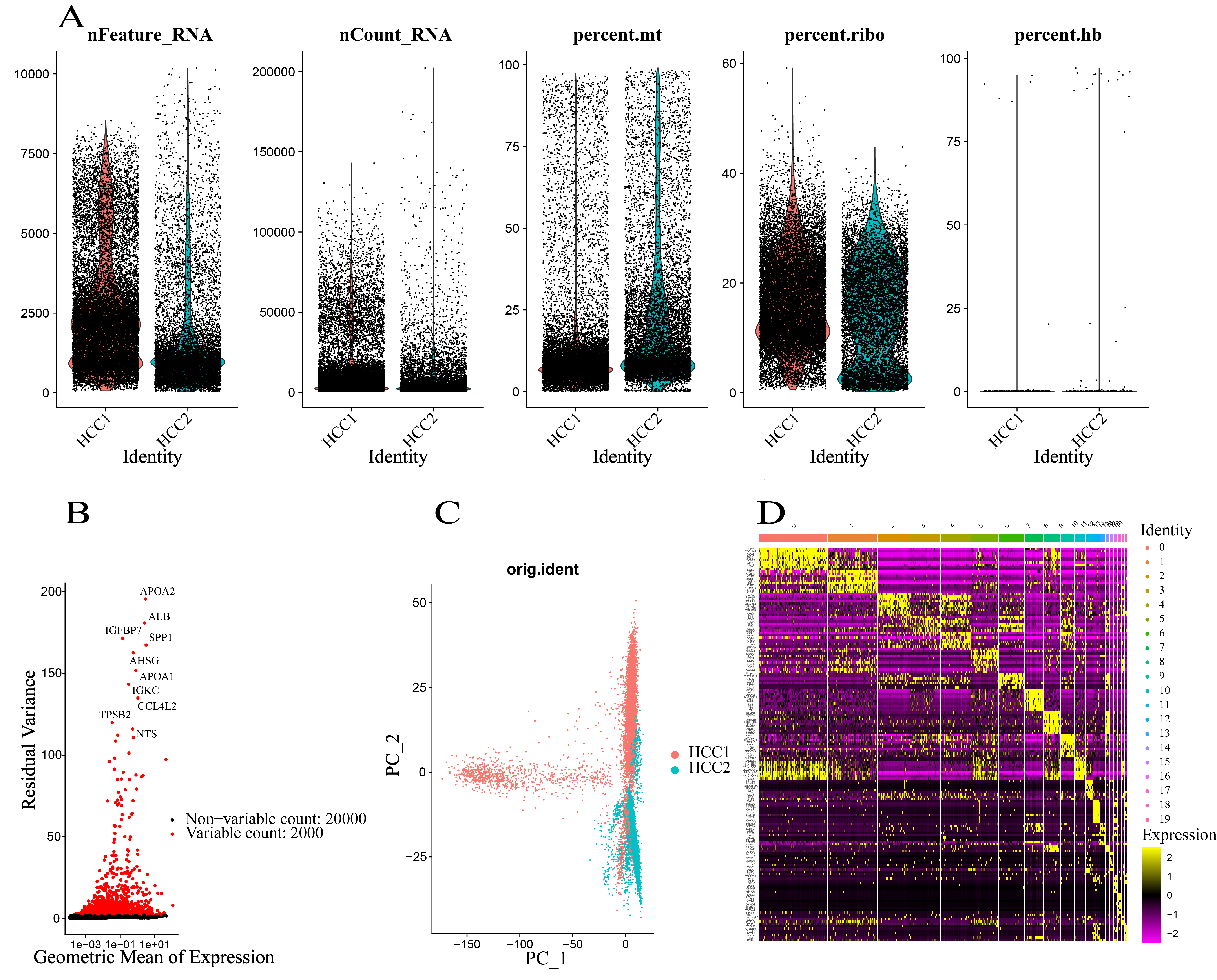

Supplement: Supplementary Figure 3 — Quality control of single-cell sequencing data. (A) Gene expression levels in each cell of the 2 samples were in the range of 250-5000, and the percentage of mitochondrial, ribosome, and hemoglobin genes was controlled below 15%. (B) We chose 2000 hypervariable genes from all of the genes, which are in red, and tagged the first ten. (C) Reduced-dimensional representations of the cells are visualized in the scatter plot. (D) After dimension reduction through PCA, we found that the cells were clustered into 20 clusters. [file Image_3.jpeg]

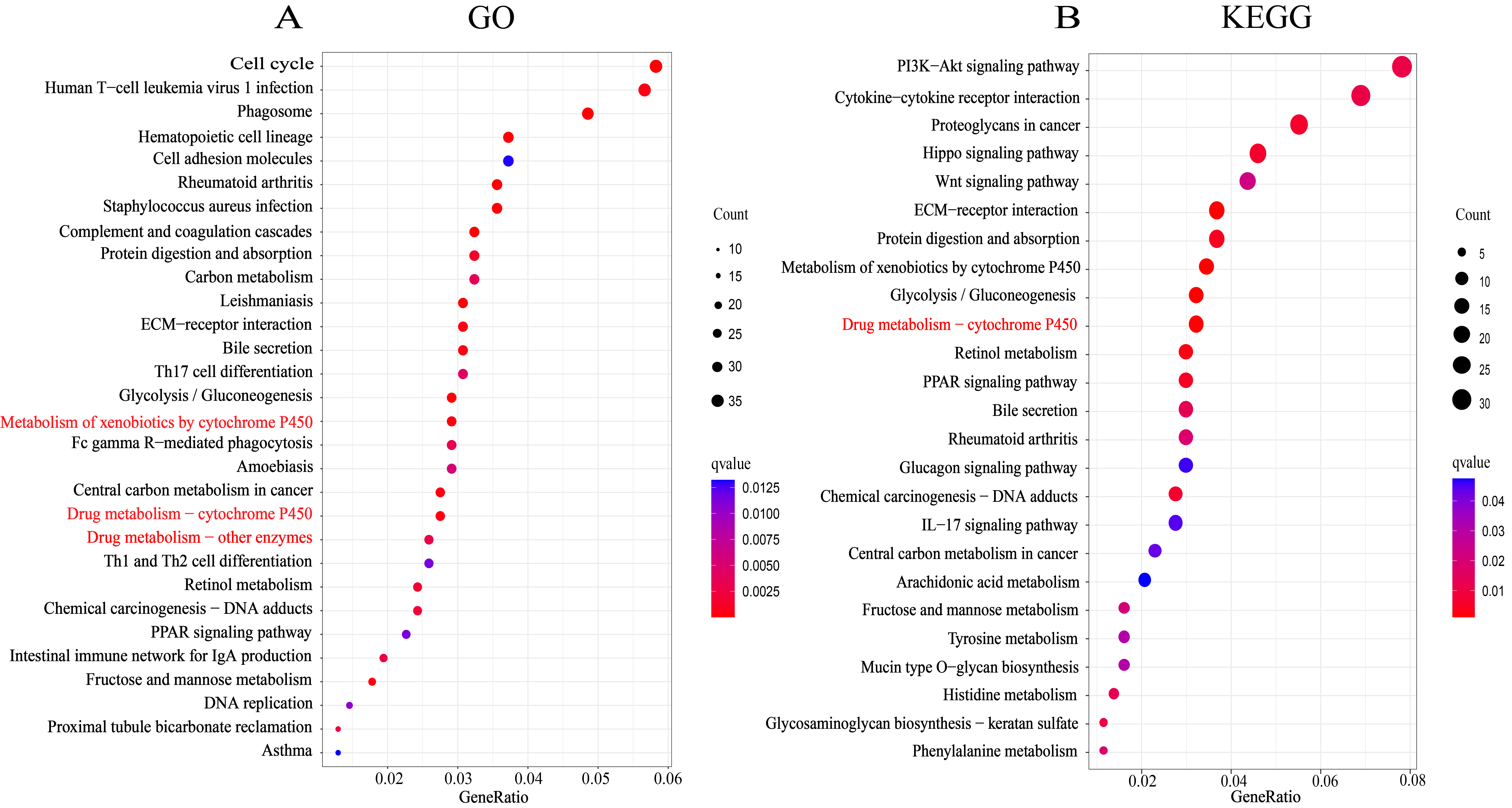

Supplement: Supplementary Figure 4 — Enrichment analysis of differentially expressed genes in the high- and low-risk group in the TCGA cohort. (A, B) In GO and KEGG analyses we found that these genes were mainly associated with drug metabolism pathway. [file Image_4.jpeg]
