# Supplementary material for: Identification of the mitophagy-related diagnostic biomarkers in hepatocellular carcinoma based on machine learning algorithm and construction of prognostic model
Source: Front Oncol. 2023 Mar 1;13:1132559. doi: 10.3389/fonc.2023.1132559 (PMC10014545; doi:10.3389/fonc.2023.1132559)
Supplement: Supplementary file 5 [file Table_1.docx]

**Supplementary Table 1** List of the specific primers for 10 prognostic Gene

| Gene | Forward | Reverse |
| --- | --- | --- |
| G6PD | 5’- CGAGGCCGTCACCAAGAAC -3’ | 5’- GTAGTGGTCGATGCGGTAGA -3’ |
| KIF20A | 5’- CAA​GAG​GCA​GAC​TTT​GCG​GCT​A -3’ | 5’- GCT​CTG​GTT​CTT​ACG​ACC​CAC​T -3’ |
| SLC1A5 | 5’- CAACCTGGTGTCAGCAGCCTT -3’ | 5’- GCACCGTCCATGTTGACGGTG -3’ |
| TPX2 | 5’- TTCAAGGCTCGTCCAAACACCG -3’ | 5’- GCTCTCTTCTCAGTAGCCAGCT -3’ |
| ANXA10 | 5’- GTCCTATGGGAAGCCTGTCA -3’ | 5’- GCACCATTCAACTCCTCGCTTTC -3’ |
| TRNP1 | 5’- CCAACTCCGACCTTGACTCC -3’ | 5’- AGCTCGGCCGCCAAGAAAA -3’ |
| ADH4 | 5’- CCCCCAAGGCTCATGAAGTT -3’ | 5’- CCACACCTCCTAGGCCAAAG-3’ |
| CYP2C9 | 5’- TGGATGAAGGTGGCAATTTT -3’ | 5’- GGGCTTCTCCCA CACAAAT -3’ |
| CFHR3 | 5′-AGTCCGTCAGACCACAGTTAC -3′ | 5’-CTGCTGTTGCATATCCTGGTTTA -3′ |
| SPP1 | 5’- CGAGGTGATAGTGTGGTTTATGG -3’ | 5’- GCACCATTCAACTCCTCGCTTTC -3’ |
| GAPDH | 5’- TGTGGGCATCAATGGATTTGG -3’ | 5’- ACACCATGTATTCCGGGTCAAT -3’ |
