# Supplementary material for: Identification of the mitophagy-related diagnostic biomarkers in hepatocellular carcinoma based on machine learning algorithm and construction of prognostic model
Source: Front Oncol. 2023 Mar 1;13:1132559. doi: 10.3389/fonc.2023.1132559 (PMC10014545; doi:10.3389/fonc.2023.1132559)
Supplement: Supplementary file 6 [file Table_2.docx]

**Supplementary Table 2** List of 226 differential genes between cluster A and cluster B patterns

| ID | gene | | ID | | gene | | ID | gene | ID | gene |
| --- | --- | --- | --- | --- | --- | --- | --- | --- | --- | --- |
| 1 | HMGA1 | | 41 | | CCNB2 | | 81 | ACMSD | 121 | SLC6A8 |
| 2 | BOP1 | | 42 | | C8A | | 82 | EPS8L3 | 122 | OGDHL |
| 3 | CLIC1 | | 43 | | NPM3 | | 83 | HAO1 | 123 | HSD17B13 |
| 4 | SNHG6 | | 44 | | KIF20A | | 84 | THRSP | 124 | ANXA10 |
| 5 | RECQL4 | | 45 | | ACSL1 | | 85 | TNFRSF21 | 125 | MMP11 |
| 6 | GLYATL1 | | 46 | | NR1I2 | | 86 | SLC28A1 | 126 | CDO1 |
| 7 | TTLL4 | | 47 | | CYP2A6 | | 87 | C6 | 127 | CFHR4 |
| 8 | G6PD | | 48 | | GLYAT | | 88 | CYP2C8 | 128 | AFM |
| 9 | AURKB | | 49 | | PCK2 | | 89 | KMO | 129 | MDK |
| 10 | TAT | | 50 | | LAPTM4B | | 90 | SLC38A4 | 130 | DNASE1L3 |
| 11 | KIFC1 | | 51 | | F9 | | 91 | DMKN | 131 | PITX1 |
| 12 | ABAT | | 52 | | CYP2A7 | | 92 | FETUB | 132 | ALPL |
| 13 | PTTG1 | | 53 | | FBP1 | | 93 | ACSM5 | 133 | MAPK13 |
| 14 | CYP8B1 | | 54 | | CYP4F2 | | 94 | NT5DC2 | 134 | C8B |
| 15 | GBA3 | | 55 | | SLC10A1 | | 95 | DMGDH | 135 | CPS1 |
| 16 | MOGAT2 | | 56 | | BAMBI | | 96 | CYP4A22 | 136 | OTC |
| 17 | HJURP | | 57 | | TEAD2 | | 97 | RTP3 | 137 | TESC |
| 18 | CCNB1 | | 58 | | TMSB10 | | 98 | ABCB4 | 138 | RELN |
| 19 | ACSM2A | | 59 | | XDH | | 99 | ANG | 139 | BHMT |
| 20 | PAFAH1B3 | | 60 | | ETV4 | | 100 | UGT2B10 | 140 | TRNP1 |
| 21 | KIF2C | | 61 | | AGXT2 | | 101 | PYCR1 | 141 | SAA4 |
| 22 | GPD1 | | 62 | | NAT2 | | 102 | PLG | 142 | GCGR |
| 23 | MARCKSL1 | | 63 | | SLC1A5 | | 103 | AASS | 143 | AZGP1 |
| 24 | TRIP13 | | 64 | | RDH16 | | 104 | ADH1B | 144 | ADH4 |
| 25 | BIRC5 | | 65 | | F11 | | 105 | TOP2A | 145 | AKR7A3 |
| 26 | SLC22A1 | | 66 | | NDRG1 | | 106 | ACOX2 | 146 | UGT2B7 |
| 27 | SPHK1 | | 67 | | TPX2 | | 107 | SOX9 | 147 | CA9 |
| 28 | HAO2 | | 68 | | DAO | | 108 | ARID3A | 148 | HPD |
| 29 | TTC36 | | 69 | | PROZ | | 109 | SEC14L2 | 149 | DPYS |
| 30 | CENPM | | 70 | | HP | | 110 | APOC3 | 150 | APOA5 |
| 31 | SLC46A3 | | 71 | | ACSM2B | | 111 | GAL3ST1 | 151 | TMEM82 |
| 32 | CDC20 | | 72 | | PFKFB1 | | 112 | GBP7 | 152 | CD24 |
| 33 | SOX4 | | 73 | | ALDOB | | 113 | CA5A | 153 | HSD11B1 |
| 34 | LRRC1 | | 74 | | CDKN3 | | 114 | BICC1 | 154 | UROC1 |
| 35 | UBE2C | | 75 | | SLC27A2 | | 115 | CTH | 155 | AFP |
| 36 | SLC27A5 | | 76 | | DBN1 | | 116 | IGF2BP2 | 156 | CFHR2 |
| 37 | MYBL2 | | 77 | | HPX | | 117 | AGXT | 157 | SLC1A1 |
| 38 | CDT1 | | 78 | | PCK1 | | 118 | HSD17B6 | 158 | DUSP9 |
| 39 | CDCA5 | | 79 | | GHR | | 119 | FNDC5 | 159 | HPR |
| 40 | ALDH8A1 | | 80 | | CYP2B6 | | 120 | RBP5 | 160 | CTNND2 |
| **Supplementary Table 2** List of 226 differential genes between cluster A and cluster B patterns | | | | | | | | | | |
| ID | gene | ID | | gene | |  |  |  |  |  |
| 161 | PON1 | 201 | | SPP1 | |  |  |  |  |  |
| 162 | SLC22A7 | 202 | | SAA1 | |  |  |  |  |  |
| 163 | ABCB11 | 203 | | SULT2A1 | |  |  |  |  |  |
| 164 | SERPINC1 | 204 | | EPCAM | |  |  |  |  |  |
| 165 | APOF | 205 | | AP1M2 | |  |  |  |  |  |
| 166 | SDS | 206 | | SLC13A5 | |  |  |  |  |  |
| 167 | CCL16 | 207 | | FAM3B | |  |  |  |  |  |
| 168 | NR1I3 | 208 | | SPP2 | |  |  |  |  |  |
| 169 | CYP2C9 | 209 | | SFN | |  |  |  |  |  |
| 170 | HRG | 210 | | S100P | |  |  |  |  |  |
| 171 | FAM99A | 211 | | CFHR5 | |  |  |  |  |  |
| 172 | AKR1D1 | 212 | | LECT2 | |  |  |  |  |  |
| 173 | SYT7 | 213 | | CYP7A1 | |  |  |  |  |  |
| 174 | SPINT1 | 214 | | LYZ | |  |  |  |  |  |
| 175 | CCL20 | 215 | | ACSL4 | |  |  |  |  |  |
| 176 | VIL1 | 216 | | SAA2 | |  |  |  |  |  |
| 177 | GREM2 | 217 | | C9 | |  |  |  |  |  |
| 178 | SLC29A4 | 218 | | LOXL4 | |  |  |  |  |  |
| 179 | BBOX1 | 219 | | MT1X | |  |  |  |  |  |
| 180 | FMO3 | 220 | | HPGD | |  |  |  |  |  |
| 181 | GPC3 | 221 | | CYP3A4 | |  |  |  |  |  |
| 182 | DKK1 | 222 | | MT1E | |  |  |  |  |  |
| 183 | CFHR3 | 223 | | GPR88 | |  |  |  |  |  |
| 184 | TDO2 | 224 | | GSTA2 | |  |  |  |  |  |
| 185 | ARG1 | 225 | | NQO1 | |  |  |  |  |  |
| 186 | GNMT | 226 | | LCN2 | |  |  |  |  |  |
| 187 | UPB1 |  | |  | |  |  |  |  |  |
| 188 | SERPINA11 |  | |  | |  |  |  |  |  |
| 189 | MASP2 |  | |  | |  |  |  |  |  |
| 190 | ADH1C |  | |  | |  |  |  |  |  |
| 191 | DCDC2 |  | |  | |  |  |  |  |  |
| 192 | BEX2 |  | |  | |  |  |  |  |  |
| 193 | CYP4A11 |  | |  | |  |  |  |  |  |
| 194 | PGC |  | |  | |  |  |  |  |  |
| 195 | KRT19 |  | |  | |  |  |  |  |  |
| 196 | G6PC |  | |  | |  |  |  |  |  |
| 197 | PGLYRP2 |  | |  | |  |  |  |  |  |
| 198 | AQP9 |  | |  | |  |  |  |  |  |
| 199 | PEG10 |  | |  | |  |  |  |  |  |
| 200 | TTR |  | |  | |  |  |  |  |  |
