# Supplementary material for: Identification of the mitophagy-related diagnostic biomarkers in hepatocellular carcinoma based on machine learning algorithm and construction of prognostic model
Source: Front Oncol. 2023 Mar 1;13:1132559. doi: 10.3389/fonc.2023.1132559 (PMC10014545; doi:10.3389/fonc.2023.1132559)
Supplement: Supplementary file 7 [file Table_3.docx]

**Supplementary Table 3** List of the 145 genes were related to the prognosis of HCC

| gene | HR | HR.95L | HR.95H | pvalue |
| --- | --- | --- | --- | --- |
| G6PD | 1.385378 | 1.247683 | 1.53827 | 1.04E-09 |
| TRIP13 | 1.484125 | 1.294179 | 1.70195 | 1.60E-08 |
| KIF2C | 1.448179 | 1.27089 | 1.650199 | 2.73E-08 |
| KIF20A | 1.466349 | 1.276096 | 1.684966 | 6.72E-08 |
| CDC20 | 1.347262 | 1.208393 | 1.502089 | 7.85E-08 |
| SLC1A5 | 1.321955 | 1.193849 | 1.463807 | 8.01E-08 |
| TPX2 | 1.39621 | 1.228582 | 1.58671 | 3.14E-07 |
| HJURP | 1.449663 | 1.255894 | 1.673328 | 3.93E-07 |
| CCNB1 | 1.398454 | 1.222446 | 1.599803 | 1.03E-06 |
| SPP1 | 1.132362 | 1.075447 | 1.19229 | 2.31E-06 |
| PTTG1 | 1.351744 | 1.191824 | 1.533122 | 2.71E-06 |
| MYBL2 | 1.255749 | 1.141129 | 1.381883 | 3.11E-06 |
| BIRC5 | 1.318239 | 1.173366 | 1.480999 | 3.29E-06 |
| TRNP1 | 1.222718 | 1.118968 | 1.336089 | 8.80E-06 |
| KIFC1 | 1.316202 | 1.164322 | 1.487894 | 1.12E-05 |
| CDCA5 | 1.374605 | 1.192646 | 1.584325 | 1.12E-05 |
| MARCKSL1 | 1.348123 | 1.179248 | 1.54118 | 1.22E-05 |
| UBE2C | 1.281044 | 1.146435 | 1.431458 | 1.23E-05 |
| ADH4 | 0.894756 | 0.850785 | 0.941 | 1.52E-05 |
| HMGA1 | 1.341185 | 1.173882 | 1.532332 | 1.57E-05 |
| NT5DC2 | 1.309291 | 1.156767 | 1.481926 | 2.00E-05 |
| CDT1 | 1.309305 | 1.155935 | 1.483025 | 2.24E-05 |
| CENPM | 1.298194 | 1.148828 | 1.466979 | 2.86E-05 |
| LAPTM4B | 1.304033 | 1.151534 | 1.476726 | 2.87E-05 |
| NDRG1 | 1.298941 | 1.145908 | 1.472411 | 4.32E-05 |
| DNASE1L3 | 0.782078 | 0.694321 | 0.880928 | 5.17E-05 |
| PON1 | 0.870912 | 0.81436 | 0.931391 | 5.46E-05 |
| CFHR4 | 0.843333 | 0.776206 | 0.916266 | 5.66E-05 |
| RECQL4 | 1.32382 | 1.153312 | 1.519537 | 6.68E-05 |
| TOP2A | 1.243465 | 1.116289 | 1.385131 | 7.55E-05 |
| AURKB | 1.286209 | 1.13532 | 1.457152 | 7.71E-05 |
| CYP2C9 | 0.877174 | 0.821488 | 0.936635 | 9.00E-05 |
| ANXA10 | 0.832693 | 0.758684 | 0.913921 | 0.000116 |
| CFHR3 | 0.867241 | 0.805607 | 0.93359 | 0.000153 |
| SPP2 | 0.890195 | 0.837262 | 0.946474 | 0.0002 |
| CCNB2 | 1.279159 | 1.122412 | 1.457796 | 0.000223 |
| CLIC1 | 1.351567 | 1.150926 | 1.587185 | 0.000238 |
| BAMBI | 1.216016 | 1.0941 | 1.351517 | 0.000285 |
| SFN | 1.141958 | 1.062927 | 1.226866 | 0.000286 |
| LECT2 | 0.875082 | 0.813982 | 0.940768 | 0.000302 |
| BOP1 | 1.324491 | 1.1354 | 1.545072 | 0.000349 |
| CDKN3 | 1.261342 | 1.110393 | 1.432811 | 0.000357 |
| EPS8L3 | 1.179866 | 1.076792 | 1.292807 | 0.000391 |
| G6PC | 0.879838 | 0.819535 | 0.944579 | 0.000409 |
| SLC10A1 | 0.898304 | 0.846373 | 0.953421 | 0.000416 |
| ETV4 | 1.162403 | 1.067813 | 1.265373 | 0.000511 |
| HPX | 0.881588 | 0.820365 | 0.94738 | 0.000599 |
| AFM | 0.881663 | 0.819991 | 0.947974 | 0.000664 |
| CA9 | 1.114461 | 1.046814 | 1.18648 | 0.000694 |
| SLC22A1 | 0.903811 | 0.852483 | 0.958231 | 0.000698 |
| CPS1 | 0.909359 | 0.860423 | 0.961078 | 0.000761 |
| TTLL4 | 1.281069 | 1.107516 | 1.481819 | 0.000853 |
| RDH16 | 0.885005 | 0.823498 | 0.951105 | 0.000887 |
| RTP3 | 0.891428 | 0.832499 | 0.954529 | 0.000989 |
| PAFAH1B3 | 1.214232 | 1.079343 | 1.36598 | 0.001235 |
| UPB1 | 0.872348 | 0.802891 | 0.947814 | 0.001255 |
| GLYATL1 | 0.861358 | 0.786416 | 0.943442 | 0.001311 |
| SLC27A5 | 0.869011 | 0.79679 | 0.947778 | 0.001516 |
| TAT | 0.912853 | 0.862093 | 0.966602 | 0.001786 |
| NQO1 | 1.096374 | 1.034512 | 1.161935 | 0.001903 |
| SLC22A7 | 0.901514 | 0.84434 | 0.96256 | 0.001926 |
| ADH1B | 0.895798 | 0.835369 | 0.960598 | 0.002014 |
| GHR | 0.83052 | 0.737928 | 0.934731 | 0.002076 |
| F11 | 0.839352 | 0.749909 | 0.939463 | 0.002318 |
| OGDHL | 0.868071 | 0.790324 | 0.953466 | 0.003123 |
| DMGDH | 0.854618 | 0.76917 | 0.949558 | 0.003467 |
| C6 | 0.878748 | 0.805763 | 0.958343 | 0.003481 |
| HRG | 0.922049 | 0.873157 | 0.973677 | 0.003505 |
| ANG | 0.850082 | 0.761773 | 0.948627 | 0.003703 |
| ADH1C | 0.920357 | 0.870123 | 0.973491 | 0.003754 |
| CFHR2 | 0.895511 | 0.8304 | 0.965727 | 0.004164 |
| FMO3 | 0.900059 | 0.837467 | 0.967328 | 0.004194 |
| TEAD2 | 1.216349 | 1.063681 | 1.390931 | 0.004208 |
| LRRC1 | 1.220488 | 1.06475 | 1.399005 | 0.004226 |
| MASP2 | 0.89422 | 0.828221 | 0.965479 | 0.004263 |
| SEC14L2 | 0.863518 | 0.780772 | 0.955032 | 0.004301 |
| CYP7A1 | 0.910321 | 0.853405 | 0.971033 | 0.00434 |
| TTC36 | 0.865395 | 0.783083 | 0.956359 | 0.004583 |
| ACSM2A | 0.883376 | 0.81029 | 0.963053 | 0.004887 |
| SOX4 | 1.162461 | 1.045194 | 1.292884 | 0.005525 |
| TNFRSF21 | 1.180915 | 1.049798 | 1.328409 | 0.005618 |
| PYCR1 | 1.137052 | 1.037797 | 1.245799 | 0.00585 |
| DBN1 | 1.180465 | 1.048808 | 1.328648 | 0.005963 |
| HSD17B13 | 0.925542 | 0.875671 | 0.978253 | 0.006181 |
| CD24 | 1.108596 | 1.029612 | 1.193639 | 0.006261 |
| HAO1 | 0.893219 | 0.823298 | 0.969079 | 0.006624 |
| CCL20 | 1.106803 | 1.028056 | 1.191582 | 0.007044 |
| AKR7A3 | 0.901754 | 0.836223 | 0.97242 | 0.00722 |
| AGXT | 0.906414 | 0.843696 | 0.973795 | 0.007236 |
| AKR1D1 | 0.903982 | 0.839415 | 0.973516 | 0.007588 |
| IGF2BP2 | 1.149478 | 1.037013 | 1.274139 | 0.008006 |
| HSD17B6 | 0.909239 | 0.847226 | 0.975791 | 0.008293 |
| TMSB10 | 1.155702 | 1.03683 | 1.288202 | 0.008973 |
| ALDOB | 0.920489 | 0.864258 | 0.980378 | 0.009991 |
| GNMT | 0.910744 | 0.847966 | 0.97817 | 0.010298 |
| ALDH8A1 | 0.884221 | 0.80438 | 0.971986 | 0.010821 |
| AP1M2 | 1.092787 | 1.020309 | 1.170413 | 0.011271 |
| ARID3A | 1.158488 | 1.033771 | 1.29825 | 0.011358 |
| CDO1 | 0.905182 | 0.837847 | 0.977928 | 0.011541 |
| BHMT | 0.917161 | 0.85742 | 0.981064 | 0.011861 |
| C8B | 0.891436 | 0.814616 | 0.9755 | 0.012439 |
| NR1I2 | 0.878501 | 0.793265 | 0.972896 | 0.012859 |
| SNHG6 | 1.21196 | 1.04144 | 1.410401 | 0.012963 |
| PITX1 | 1.134458 | 1.026978 | 1.253187 | 0.012986 |
| SULT2A1 | 0.924011 | 0.867965 | 0.983676 | 0.013305 |
| AQP9 | 0.92231 | 0.865045 | 0.983366 | 0.013404 |
| C8A | 0.89933 | 0.826495 | 0.978582 | 0.013801 |
| APOC3 | 0.921724 | 0.863215 | 0.984198 | 0.014851 |
| SPHK1 | 1.110732 | 1.01998 | 1.209558 | 0.015741 |
| PGLYRP2 | 0.912616 | 0.847314 | 0.98295 | 0.015781 |
| PROZ | 0.882608 | 0.796522 | 0.977997 | 0.017085 |
| CA5A | 0.881885 | 0.795043 | 0.978212 | 0.01748 |
| ABAT | 0.883383 | 0.797099 | 0.979007 | 0.018052 |
| CYP8B1 | 0.936498 | 0.88672 | 0.989071 | 0.018557 |
| PLG | 0.910618 | 0.84146 | 0.985461 | 0.020158 |
| FBP1 | 0.902552 | 0.827703 | 0.98417 | 0.020275 |
| SERPINC1 | 0.92899 | 0.872909 | 0.988675 | 0.020424 |
| S100P | 1.058065 | 1.008008 | 1.110608 | 0.022459 |
| MOGAT2 | 0.905991 | 0.832278 | 0.986232 | 0.022599 |
| UROC1 | 0.918418 | 0.853584 | 0.988177 | 0.022705 |
| FAM99A | 0.910673 | 0.83992 | 0.987385 | 0.023352 |
| CYP3A4 | 0.947224 | 0.90379 | 0.992745 | 0.023574 |
| APOF | 0.918797 | 0.85337 | 0.989241 | 0.024642 |
| SOX9 | 1.117042 | 1.013944 | 1.230622 | 0.025075 |
| SLC6A8 | 1.10834 | 1.01268 | 1.213036 | 0.025512 |
| MDK | 1.110122 | 1.012159 | 1.217565 | 0.026666 |
| PCK1 | 0.928416 | 0.869386 | 0.991455 | 0.026692 |
| CYP2C8 | 0.929041 | 0.870246 | 0.991809 | 0.027347 |
| AASS | 0.881944 | 0.788489 | 0.986476 | 0.027933 |
| BICC1 | 1.109504 | 1.008539 | 1.220578 | 0.032791 |
| F9 | 0.928713 | 0.86769 | 0.994027 | 0.032948 |
| CFHR5 | 0.933105 | 0.875545 | 0.994449 | 0.033064 |
| DUSP9 | 1.092259 | 1.006589 | 1.185221 | 0.034213 |
| TTR | 0.927538 | 0.865088 | 0.994496 | 0.034417 |
| HPD | 0.942842 | 0.891633 | 0.996993 | 0.038861 |
| AZGP1 | 0.911753 | 0.835002 | 0.995559 | 0.039478 |
| DAO | 0.903235 | 0.819826 | 0.99513 | 0.039521 |
| GBP7 | 0.911475 | 0.833839 | 0.996339 | 0.041279 |
| CTH | 0.909869 | 0.83084 | 0.996415 | 0.041606 |
| GLYAT | 0.932754 | 0.872107 | 0.997618 | 0.042408 |
| CYP4A11 | 0.920978 | 0.850512 | 0.997283 | 0.042666 |
| SLC38A4 | 0.916991 | 0.843274 | 0.997153 | 0.042699 |
| ALPL | 0.900066 | 0.812007 | 0.997675 | 0.045041 |
| HPR | 0.926315 | 0.858765 | 0.999179 | 0.047566 |
| SLC29A4 | 1.092317 | 1.000569 | 1.192477 | 0.048534 |
