# Supplementary material for: Identification of the mitophagy-related diagnostic biomarkers in hepatocellular carcinoma based on machine learning algorithm and construction of prognostic model
Source: Front Oncol. 2023 Mar 1;13:1132559. doi: 10.3389/fonc.2023.1132559 (PMC10014545; doi:10.3389/fonc.2023.1132559)
Supplement: Supplementary file 9 [file Table_5.docx]

**List Of Abbreviations**

| **Abbreviation** | **Description** |
| --- | --- |
| AUC | area under the curve |
| CNV | Copy number variations |
| DEGs | differentially expressed genes |
| GEO | Gene Expression Omnibus |
| GSVA | Gene set variation analysis |
| HCC | hepatocellular carcinoma |
| IC50 | The half-maximal inhibitory concentration |
| ICGC | International Cancer Genome Consortium |
| ICI | immune checkpoint inhibitor |
| LASSO | Least absolute shrinkage and selection operator |
| MRGs | mitophagy-related genes |
| NMF | non-negative matrix factorization |
| OS | Overall Survival |
| PCA | principal component analysis |
| ROC | receiver operating characteristic |
| ROS | reactive oxygen species |
| SVM- RFE | support vector machine- recursive feature elimination |
| TACE | transcatheter arterial chemoembolization |
| TCGA | The Cancer Genome Atlas |
| TIDE | Tumor Immunological Dysfunction and Exclusion |
| TIME | tumor immune microenvironment |
| TMB | tumor mutation burden |
